# A phosphatidic acid‐based retrograde signaling pathway responsive to abiotic stress in Arabidopsis

**DOI:** 10.1111/tpj.71107

**Published:** 2026-09-13

**Authors:** Carrie Hiser, Ron Cook, Yosia Mugume, John E. Froehlich, Christoph Benning

**Affiliations:** ^1^ MSU‐DOE Plant Research Laboratory East Lansing Michigan 48824 USA; ^2^ Department of Biochemistry and Molecular Biology Michigan State University East Lansing Michigan 48824 USA; ^3^ Department of Plant Biology Michigan State University East Lansing Michigan 48824 USA

**Keywords:** *Arabidopsis thaliana*, phosphatidic acid, abscisic acid, mediator complex, lipid phosphate phosphatases, retrograde signaling, chloroplast, lipid signaling, abiotic stress

## Abstract

Due to their sessile nature, plants are constantly exposed to the environment and must cope with sometimes extreme changes in conditions during the day or growing season. Plants depend on photosynthesis as their primary means to generate energy and building blocks, and adverse environmental conditions can stress the photosynthetic apparatus leading to the production of toxic byproducts. In the long term, stress experienced by the chloroplast must be communicated to the nucleus to adjust the expression of genes providing robust abiotic stress resilience in a process called retrograde signaling. Here, we propose a retrograde signaling mechanism that starts with the accumulation of phosphatidic acid at the outer chloroplast membrane. A mutant of *Arabidopsis thaliana*, *lppγ lppɛ1*, disrupted in two chloroplast envelope membrane‐located phosphatidic acid phosphatases, shows reduced growth and activation of abscisic acid‐mediated abiotic stress‐response pathways, among other changes, as determined by RNA‐Seq analysis. The mutant is more resistant to freezing and osmotic stress. To identify components of the proposed retrograde signaling pathway, we conducted a suppressor screen in the *lppγ lppɛ1* mutant and identified a mutation that causes the loss of MED16, which is a component of Mediator, a transcriptional complex in the nucleus affecting the expression of genes involved in abiotic stress tolerance, among others. Based on these findings, we are proposing a lipid‐based, retrograde signaling mechanism in response to abiotic stresses such as freezing.

## Significance Statement

A novel lipid‐based, retrograde abiotic stress signaling pathway is proposed based on the detailed analysis of a chloroplast phosphatidic acid mutant, *lppγ lppɛ1*, and its suppressor, *med16*.

## INTRODUCTION

Plant chloroplast lipids are mostly galactolipids instead of phospholipids (Dormann & Benning, 2002). The precursor is diacylglycerol (DAG), which is made by dephosphorylating phosphatidic acid (PA), the first lipid formed by complete acylation of glycerol‐3‐phosphate (Lavell & Benning, 2019). PA and DAG can be made by an endoplasmic reticulum (ER)‐localized pathway, and these precursors are generally used to synthesize phospholipids or triacylglycerol (Lavell & Benning, 2019). PA and DAG are also made in the chloroplast by the plastid pathway and are used to synthesize phosphatidylglycerol (PG), the sulfolipid sulfoquinovosyldiacylglycerol (SQDG), and the two galactolipids monogalactosyldiacylglycerol (MGDG) and digalactosyldiacylglycerol (DGDG) (Lavell & Benning, 2019). In *Arabidopsis thaliana* (Arabidopsis), there are three known chloroplast membrane‐associated lipid phosphate phosphatases (LPPs): LPPγ, LPPɛ1, and LPPɛ2, of which LPPγ and LPPε1 participate in dephosphorylation of ER‐derived PA (Cook et al., 2024; Nakamura et al., 2007; Nguyen & Nakamura, 2023). However, the phosphatidic acid phosphatase(s) (PAP) that dephosphorylates plastid‐derived PA for the synthesis of galactolipids in the chloroplast remains elusive (Cook et al., 2024; Nguyen & Nakamura, 2023).

Previous research in our laboratory investigated mutants in LPPγ and LPPɛ1, localized to the chloroplast outer envelope membrane, and LPPɛ2, localized to the inner envelope and/or thylakoid membranes (Cook et al., 2024). No apparent phenotypes were found for any of the single mutants or the *lppγ lppɛ2* or *lppɛ1 lppɛ2* double mutants, but the *lppγ lppɛ1* double mutant was dwarfed and showed increased anthocyanin production (Cook et al., 2024). This dwarf phenotype was not alleviated by low light or high CO_2_, suggesting that it was not due to photosynthetic stress, nor was it due to activation of the jasmonic acid (JA) or salicylic acid (SA) pathways (Cook et al., 2024). No significant differences from wild type were seen in the overall lipid composition of the *lppγ lppɛ1* mutant, nor in the flux of label from ^14^C‐acetate into PA, phosphatidylcholine, PG, MGDG, or SQDG in isolated chloroplasts (Cook et al., 2024). Overexpression of either *LPPγ* or *LPPɛ1* in the *lppγ lppɛ1* background restored plants to the normal size and did not affect lipid composition, suggesting that these LPPs have partially redundant functions (Cook et al., 2024). It was concluded that the *lppγ lppɛ1* phenotype may result from a localized accumulation of PA in which PA acts not as a bulk lipid but as a signaling molecule (Cook et al., 2024).

PA has been shown to be a signal or second messenger in plants (Kolesnikov et al., 2022; Lavell & Benning, 2019; Muthan et al., 2013; Testerink & Munnik, 2005, 2011; Yao et al., 2024). PA is predominantly produced by the action of phospholipase D on membrane lipids (Hou et al., 2016; Li et al., 2009) or it can be produced by the sequential action of phospholipase C (PLC) and diacylglycerol kinase (DAGK) (Arisz et al., 2009; Testerink & Munnik, 2011). Environmental stresses such as cold, heat, drought, salt, and other osmotic stresses, wounding, and pathogens have been reported to trigger rapid PA production, in some cases involving the response to abscisic acid (ABA) (de Jong et al., 2004; Hou et al., 2016; Laxalt et al., 2007; McLoughlin et al., 2013; Munnik et al., 2000; Zhang et al., 2004). For comprehensive reviews of PA formation and function in stress‐responses, see (Kolesnikov et al. 2022; Testerink & Munnik 2011; Wang et al. 2006). PA is involved in both ABA and auxin responses by affecting the activity and/or distribution of the involved proteins. One example in Arabidopsis is the binding of PA to the negative regulator ABI1 (ABA INSENSITIVE 1; AT4G26080), which inhibits its phosphatase activity and alters its subcellular location, thereby promoting the ABA response (Yao et al., 2024).

The Mediator complex is a large transcriptional regulatory complex which, in Arabidopsis, consists of 19–21 generally conserved subunits and six plant‐specific subunits (Chen et al., 2022; Kidd et al., 2011). In plants, the Mediator complex has been reported to be involved in many stress responses, including cold acclimation (Boyce et al., 2003; Crawford et al., 2020; Hemsley et al., 2014; Knight et al., 1999, 2009), heat stress (Crawford et al., 2020; Kim & Gross, 2013), drought and osmotic stress (Boyce et al., 2003; Knight et al., 1997; Tian et al., 2017), salt stress (Knight et al., 1997; Tian et al., 2017), defense pathways (Mao et al., 2019; Wang et al., 2015, 2016; Wathugala et al., 2012; Xue et al., 2019; Zhang et al., 2012, 2013), anthocyanin production (Dolan et al., 2017; Guo et al., 2021), circadian clock–regulated and flowering time gene expression (Knight et al., 2008), trichome development (Fornero et al., 2019), metal homeostasis in roots (Yang et al., 2014), and phosphate stress (Raya‐Gonzalez et al., 2021). Many of these Mediator‐related responses occur through ABA‐induced transcriptional responses (Chen et al., 2022; Chong et al., 2020; Guo et al., 2021; Lee et al., 2021). The Mediator complex consists of three modules (head, middle, tail) and a detachable kinase (CDK8) regulatory domain (Buendia‐Monreal & Gillmor, 2016; Chao et al., 2024; Chen et al., 2022; Freytes et al., 2024; Zhao et al., 2021). The head domain interacts with RNA polymerase II and general transcription factors (TFs) while the tail domain interacts with specific TFs (reviewed in Freytes et al., 2024), and contains subunit 16 (MED16), which has been specifically implicated in many of the aforementioned stress responses (reviewed in Buendia‐Monreal & Gillmor, 2016; Yang et al., 2016).

Here, we sought to mechanistically connect mutations in two chloroplast phosphatidic acid phosphatases to the dwarf phenotype of the double mutant *lppγ lppɛ1* (Cook et al., 2024). We found many differentially expressed genes in *lppγ lppɛ1* as compared to the wild type, many of which are related to stress responses and to the ABA signaling pathway, which could divert resources from growth to defense. As *lppγ lppɛ1* likely accumulates PA at the chloroplast envelopes (Cook et al., 2024), we hypothesized that this PA has a retrograde signaling role affecting gene expression. To begin to identify components of this proposed retrograde signaling pathway, we screened a library of EMS‐mutagenized *lppγ lppɛ1* seeds. A mutation in subunit 16 of the Mediator Complex, which is not yet known to be associated with PA signaling in plants, reversed the phenotype of *lppγ lppɛ1*.

## RESULTS

### RNA‐Seq identified differentially expressed genes in *lppγ lppɛ1* relative to wild type

We hypothesized that the growth phenotype of the *lppγ lppɛ1* mutant may have resulted from altered retrograde signaling that could affect expression of nuclear genes related to stress experienced by the chloroplast. To determine whether stress‐response genes are constitutively upregulated in the *lppγ lppɛ1* mutant, we compared gene expression in the *lppγ lppɛ1* mutant to that in the wild type grown under standard growth conditions by using RNA‐Seq. Out of 19 075 genes, the differences in the expression of 6693 genes had adjusted *P* values <0.05 and were therefore considered significant (Data S1).

**Figure 1:**
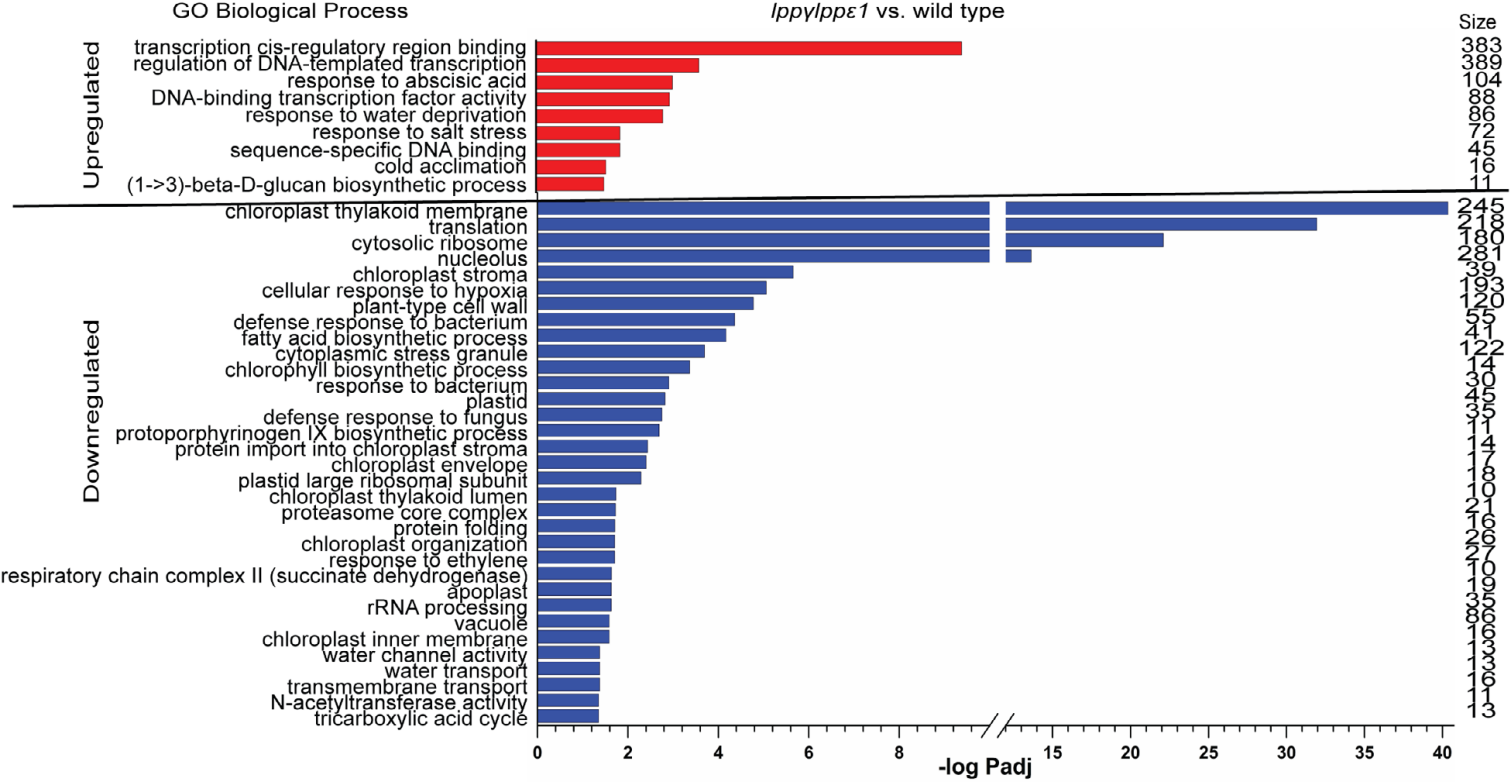
Gene Ontology (GO) term enrichment of upregulated and downregulated genes from RNA‐Seq analysis of the *lppγ lppɛ1* double mutant versus wild type.

Gene Ontology (GO) analysis (Figure 1) found upregulation in *lppγ lppɛ1* of genes involved in three transcription‐related categories plus three abiotic stress responses (cold acclimation, salt stress, and water deprivation). Also upregulated is the response to ABA, which is involved in many abiotic stress‐response pathways (Chen et al., 2020; Trivedi et al., 2016; Wei et al., 2025). Indeed, among the top 10 genes with altered expression in *lppγ lppɛ1* (Table 1) are three ABA‐response‐related genes. These upregulated genes are *LTI65/RD29B* (AT5G52300), which responds to cold, salt, and desiccation in an ABA‐dependent manner (Nordin et al., 1993), and the gene encoding a divergent histone HIS1‐3/HON3 (AT2G18050), which is upregulated by ABA and dehydration (Ascenzi & Gantt, 1997) but downregulated by salt stress (Wu et al., 2022). The gene encoding AFP3 (AT3G29575), a co‐factor that destabilizes the TF ABI5 (AT2G36270) in the ABA core signaling pathway (Du et al., 2025; Garcia et al., 2008), is strongly downregulated. Another of the top 10 upregulated genes is *SIS* (AT5G02020), whose encoded protein is involved in salt tolerance (Brinker et al., 2010). Downregulated GO categories included chloroplast membranes and organization, fatty acid synthesis, translation, response to biotic stresses (bacteria, fungi), and hypoxia.

**Table 1:** Top 10 differentially expressed genes in the *lppγ lppɛ1* mutant relative to the wild type

GeneID	Symbol	Fold change	*P*adj	Protein encoded by gene
AT1G64660	*MGL*	865	3.07E‐189	Methionine gamma‐lyase; catalyzes the degradation of methionine into methanethiol, alpha‐ketobutyrate and ammonia
AT2G41190	*AVT1A*	25.5	2.61E‐138	Transmembrane amino acid transporter family protein
AT5G52300	*LTI65*/*RD29B*	516	1.45E‐128	Low‐Temperature‐Induced 65; Responsive To Desiccation 29B; expression induced by cold, high salt, and desiccation via ABA
AT2G24600	AT2G24600	−16.1	2.49E‐126	Ankyrin repeat family protein
AT2G18050	*HIS1‐3*/*HON3*	78.6	5.49E‐116	Structurally divergent linker histone whose expression is induced by dehydration and ABA
AT1G64110	*DAA1*	865	8.53E‐113	DUO1‐activated ATPase 1; target of the male germline‐specific transcription factor DUO1
AT5G02020	*SIS*	7.98	1.71E‐112	Salt Induced Serine rich; encodes a protein involved in salt tolerance
AT3G29575	*AFP3*	9.4	2.26E‐111	ABI5 Binding Protein 3; co‐factor that destabilizes ABI5 in ABA signaling
AT2G27402	AT2G27402	−22.3	9.39E‐108	Plastid transcriptionally active protein 18
AT3G28290	*AT14A*	19.1	7.09E‐82	A protein with sequence similarity to integrins; Note that there are two AT14A genes

*P*adj is the adjusted *P* value; significant if <0.05.

There are 24 genes that are upregulated greater than 100‐fold (Table S2). These include several genes known to be upregulated by stress: *LEA7* (AT1G52690), whose product stabilizes lactate dehydrogenase during drying and freezing (Popova et al., 2015); *TSPO* (AT2G47770), encoding a widely conserved membrane protein induced by many stresses (Hiser et al., 2021); *LTI65/RD29B*; *ABR* (AT3G02480), encoding a late embryogenesis abundant (LEA) protein upregulated by ABA, NaCl and mannitol, and light deprivation (Su et al., 2016); genes for two heavy metal transport/detoxification superfamily proteins HIPP22 (AT1G22990) and HIPP24 (AT4G08570) (de Abreu‐Neto et al., 2013); and genes for two proteins involved in anthocyanin production, dihydroflavonol‐4‐reductase (AT5G42800) and MYB90/PAP2 (AT1G66390) (Gonzalez et al., 2008). There are four genes that are greater than 100‐fold downregulated (Table S2): *CRK30* (AT4G11460) and *CRK31* (AT4G11470), both encoding cysteine‐rich receptor‐like protein kinases (Wrzaczek et al., 2010); *PDF1.2/LCR77* (AT5G44420), encoding an ethylene‐ and jasmonate‐responsive plant defensin (Manners et al., 1998); and *PER4* (AT1G14540), encoding a class III peroxidase cell wall‐targeted protein (Fernandez‐Perez et al., 2015).

### Differential expression of select lipid genes

Transcripts apparently from the *LPPγ* and *LPPɛ1* genes were present but of lower abundance, reduced 5.6‐fold and 4.1‐fold, respectively. We hypothesize that shortened *LPPγ‐* and *LPPɛ1‐*derived transcripts were produced from these mutant loci and were present in the short‐read library and therefore detected by the RNA‐Seq methodology. The *LPPγ* T‐DNA insertion is within the coding sequence near the start of the gene while the *LPPɛ1* insertion is near the start of the fourth exon; in both cases, the early part of the transcript could have been produced. The expressions of other known phosphatidic acid phosphatase encoding genes (*LPPɛ2*, *LPP2*, *LPP3*, *LPPβ*, *PAH1*, and *PAH2*) were not significantly altered (*P* values >0.05) in *lppγ lppɛ1*.

Few other genes involved in lipid metabolism are differentially expressed in *lppγ lppɛ1* (Data S1). Those that are upregulated include genes encoding enzymes that can generate phosphatidic acid (PA): phospholipases PLC4 (AT5G58700) and PLC5 (AT5G58690) together with diacylglycerol kinases DGK1 (AT5G07920) and DGK7 (AT4G30340); and the phospholipases PLDγ (AT4G11850) and PLDα2 (AT1G52570) (Arisz et al., 2009; Testerink & Munnik, 2005). Also upregulated were the genes encoding fatty acid desaturases FAD2 (AT3G12120) and AAD4 (AT3G02620), the lipid transfer protein LTP3 (AT5G59320), and the diacylglycerol acyltransferase DGAT1/AS11/BX45 (AT2G19450), which catalyzes the last step in triacylglycerol synthesis (Hobbs et al., 1999). Some genes encoding PA‐binding proteins are also upregulated in *lppγ lppɛ1*: ABI2 (AT5G57050), an ABA signaling‐related protein phosphatase 2C, and the SNF1‐related protein kinases SnRk2.5 (AT5G63650) and SnRk2.7 (AT4G40010) (Testerink & Munnik, 2005). However, genes encoding other PA‐binding proteins are downregulated: MPK3 (AT3G45640), a kinase upregulated in response to stresses (Zhang & Zhang, 2022), and LHY (AT1G01060), a putative MYB‐related TF involved in circadian rhythm (Green & Tobin, 2002).

### Differential regulation of the ABA core signaling pathway in *lppγ lppɛ1*

A number of genes related to the ABA core signaling pathway are differentially expressed in *lppγ lppɛ1* relative to wild type (Table 2). These include a downregulated gene for one ABA receptor (PYL6/RCAR9; AT2G40330) and five upregulated genes encoding clade A protein phosphatases of type 2C (PP2Cs), including ABI2, HAI1/SAG113 (AT5G59220), HAI2/AIP1/AKT1/HON (AT1G07430), HAB1 (AT1G72770), and PP2CA/AHG3 (AT3G11410). Genes encoding two subgroup III, ABA‐responsive SnRK2s are also upregulated: SnRK2.6/SnRK2E/OST1 (AT4G33950) and CIPK25/SnRK3.25/SAG114 (AT5G25110). Twelve differentially expressed genes encoding TFs known to be involved in ABA responses (Table 3) were upregulated in *lppγ lppɛ1*. These include genes for: AREB1/ABF2 (AT1G45249) and AREB2/ABF4 (AT3G19290), which bind to ABA‐responsive elements (ABREs) in ABA‐regulated genes (Fujita et al., 2005; Nakashima et al., 2006); six AITR/DIL/DIG TFs that act as feedback inhibitors to ABA signaling and thereby negatively regulate response to stress (Tian et al., 2017); and ABI5 (AT2G36270), an important ABA core bZIP TF related to stress response (Ayub et al., 2025; Finkelstein & Lynch, 2000; Skubacz et al., 2016). The gene for the R2R3‐type TF MYB96 (AT5G62470), which represses the transcription of *ABI4* and some ABA biosynthesis genes (Lee et al., 2015; Lee & Seo, 2015), is the only ABA‐related TF‐encoding gene that was downregulated. More than 20 other genes known to be induced by or whose proteins bind ABA are also differentially expressed in *lppγ lppɛ1* (Data S1), including *LTI65/RD29B*, *HIS1‐3/HON3*, and *AFP3* from the top 10 differentially expressed genes (Table 1).

**Table 2:** Abscisic acid (ABA) signaling pathways genes that are differentially regulated in the *lppγ lppɛ1* mutant relative to the wild type

GeneID	Symbol	Fold change	*P*adj	Protein encoded by gene
AT2G40330	*PYL6*/*RCAR9*	−9.77	1.98E‐06	ABA receptor
AT5G59220	*HAI1*/*SAG113*	35.40	3.36E‐72	Clade A protein phosphatase 2C; negative regulator of osmotic stress and ABA signaling; interacts with SnRK2.6 and SnRK3.25
AT1G07430	*HAI2*/*AIP1*/*AKT1*/*HON*	10.1	7.04E‐26	Clade A protein phosphatase 2C; negatively regulates seed dormancy
AT4G26080	*ABI1*	1.66	0.024824335	Clade A protein phosphatase 2C; negatively regulates SnRKs 2.2,2.3,2.4,2.6; inhibited by PA
AT5G57050	*ABI2*	4.33	1.95E‐13	Clade A protein phosphatase 2C; represses SnRKs 2.2,2.3 and 2.6; likely to be inhibited by PA
AT3G11410	*PP2CA*/*AHG3*	2.06	0.000297675	Clade A protein phosphatase 2C; represses SnRK2.6, upregulated by drought and ABA; inhibited by PA
AT1G72770	*HAB1*	2.32	0.000199242	Clade A protein phosphatase 2C; represses SnRK2.6
AT5G25110	*CIPK25*/*SnRK3.25*/*SAG114*	37.40	8.80E‐38	SNF1‐related protein kinase subgroup III; induced by salt and anoxia, involved in stomatal closure
AT4G33950	*SnRK2.6*/*SnRK2E*/*OST1*	2.71	1.47E‐11	SNF1‐related protein kinase subgroup III; calcium‐independent, activated by ABA, salt stress, osmotic stress, involved in stomatal closure

*P*adj is the adjusted *P* value; significant if <0.05.

**Table 3:** Abscisic acid (ABA)‐associated transcription factors that are differentially expressed in the *lppγ lppɛ1* mutant relative to the wild type

GeneID	Symbol	Fold change	*P*adj	Protein encoded by gene
AT1G54160	*NF‐YA5*	11.3	9.38E‐68	Member of the CCAAT‐binding TF family; upregulated by ABA and drought
AT1G51140	*FBH3*	6.93	4.69E‐31	Basic helix–loop–helix TF involved in photoperiodism flowering; DNA‐binding inhibited by ABA
AT5G63350	*AITR6*/*DIL1*/*SDR4*/*SFL3*	15.1	4.23E‐28	ABA‐induced transcription repressors that act as feedback regulators in ABA signaling
AT3G48510	*AITR2*/*DIG1*	34.4	6.16E‐24	
AT5G40790	*AITR3*/*DIL2*	115	1.81E‐17	
AT3G27250	*AITR1*/*DIL4*/*SLF6*	16.3	9.25E‐17	
AT5G50360	*AITR5*/*DIG2*	48.2	9.78E‐15	
AT5G40800	*AITR4*/*DIL3*	12.2	3.16E‐14	
AT2G36270	*ABI5*/*DBPF1*	16.7	1.50E‐16	bZIP TF involved in ABA signaling during seed maturation and germination; regulates a subset of LEA genes; a direct target gene of ABF3 under salt stress
AT4G34000	*ABF3*	3.44	1.71E‐13	bZIP TF expressed in response to stress and ABA
AT3G19290	*ABF4*/*AREB2*	2.11	2.30E‐10	bZIP TF with specificity for ABREs; mediates ABA‐dependent stress responses through SnRK2 pathway
AT1G45249	*ABF2*/*AREB1*	2.66	3.78E‐08	bZIP TF that binds to the ABREs of ABA‐inducible genes. Enhances drought tolerance and required for normal glucose response. Expressed constitutively in roots, leaf vascular tissues, and hydathodes or in all tissues under stress conditions
AT5G62470	*MYB96*	−2.93	0.00043348	Myb family TF; decreases ABI4 and some ABA synthesis gene transcription

*P*adj is the adjusted *P* value; significant if <0.05.

### Differential expression of cold and drought regulated genes in *lppγ lppɛ1*

**Figure 2:**
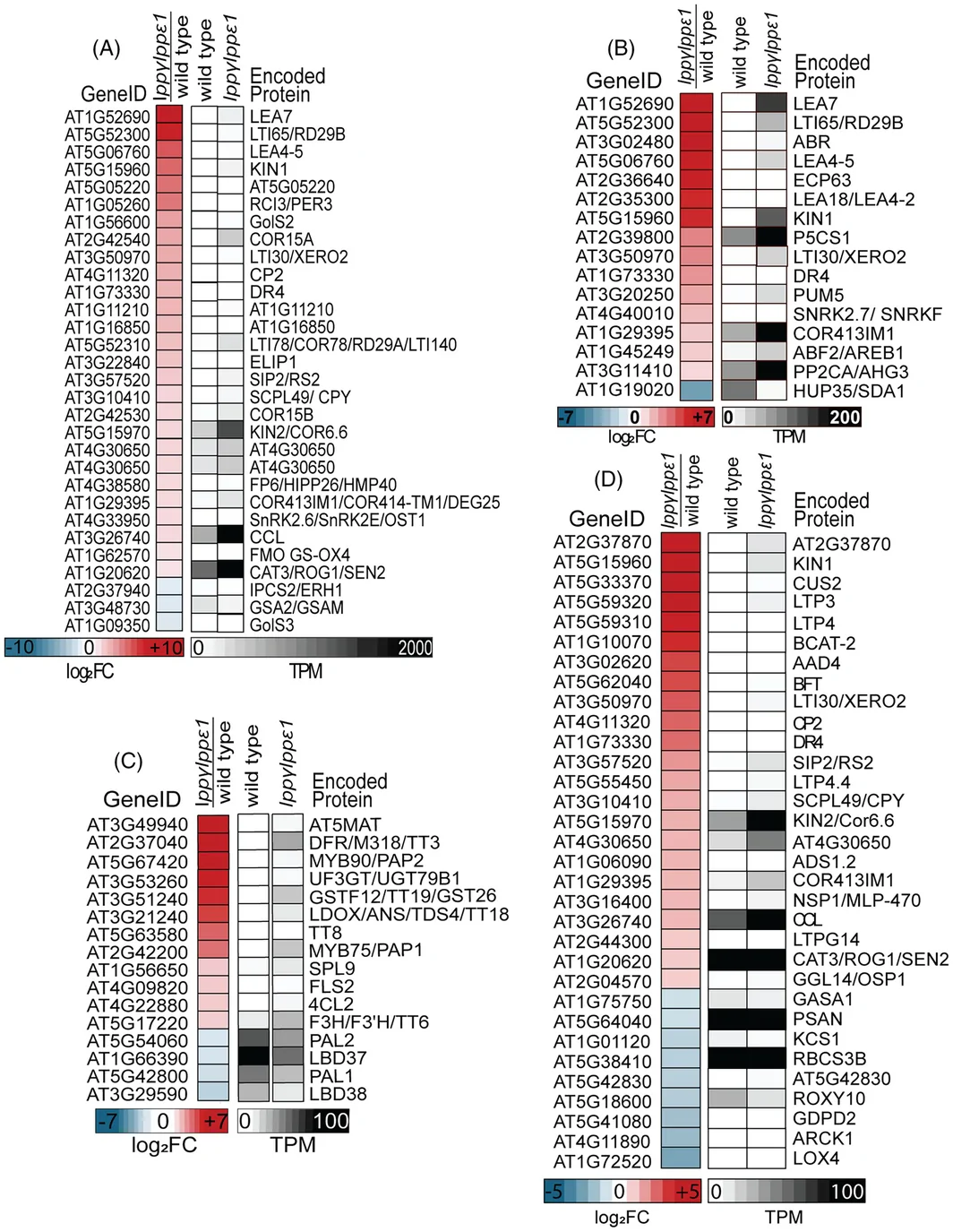
Genes that are differentially expressed in *lppγ lppɛ1* relative to the Col‐0 wild type. (A) Cold stress related genes. (B) Drought and osmotic stress related genes. (C) Anthocyanin biosynthesis and regulatory genes. (D) MED16‐influenced genes.

At least 30 cold stress‐induced genes, as listed in Boyce et al. (2003) and Hemsley et al. (2014), are upregulated in *lppγ lppɛ1* (Figure 2A), including the ABA‐inducible *LTI65/RD29B* gene from the top 10 differentially expressed genes (Table 1). Other ABA‐inducible cold stress genes include those encoding: KIN1 (AT5G15960), a possible antifreeze protein (Kurkela & Borg‐Franck, 1992; Lee et al., 2005; Wang et al., 1995); KIN2 (AT5G15970), a protein whose gene is induced by cold and ABA and which may be involved in cold acclimation and salt tolerance (Hemsley et al., 2014; Kurkela & Borg‐Franck, 1992; Wang et al., 1995); LTI30/XERO2 (AT3G50970), a dehydrin (Gupta et al., 2019); COR413‐TM1/COR414‐TM1/DEG25 (AT1G29395), a chloroplast integral membrane protein whose gene expression is upregulated by many stresses (Lee et al., 2005; Okawa et al., 2008); and SnRK2.6/SnRK2E/OST1 (AT4G33950), a Ca‐dependent SnRK2 protein kinase involved in the ABA core signaling pathway (Fujita et al., 2009; Hasan et al., 2022; Kulik et al., 2011; Nakashima et al., 2009).

Although several of the cold‐responsive genes are also upregulated by water deprivation, osmotic stress, or salt stress, there are also genes that appear to be exclusively related to drought and osmotic stress (Figure 2B). These include genes encoding SnRK2.7/SnRKF (AT4G40010), a subgroup II SnRK2 that is activated by osmotic stress but only slightly by ABA (Fujii et al., 2011; Kulik et al., 2011; Mizoguchi et al., 2010), and additional members of the LEA protein family which accumulate in response to low water availability during development or stress (Candat et al., 2014; Olvera‐Carrillo et al., 2010). Also upregulated are genes *HIS1‐3/HON3*, one of the top 10 upregulated genes (Table 1) and reported to be induced by drought stress (Ascenzi & Gantt, 1997) but downregulated by salt stress (Wu et al., 2022); and *P5CS1 (AT2G39800)*, encoding delta1‐pyrroline‐5‐carboxylate synthase that catalyzes the rate‐limiting step in proline synthesis and its gene is highly upregulated by drought and salinity (Knight et al., 1997).

### Increased expression of genes involved in anthocyanin biosynthesis in *lppγ lppɛ1*

Some genes involved in anthocyanin production are differentially expressed in *lppγ lppɛ1* relative to wild type (Figure 2C). Expressions of the genes for enzymes in the general phenypropanoid pathway and early anthocyanin biosynthesis are all changed by 2.5‐fold or less. However, two genes for enzymes in the late anthocyanin biosynthesis main pathway are strongly upregulated: the dihydroflavonol‐4‐reductase (DFR; AT5G42800) gene is 245‐fold upregulated and the anthocyanidin synthase/leucoanthocyanidin dioxygenase (ANS/LDOX/TDS4/TT18; AT4G22880) gene is 37.9‐fold upregulated. Two genes encoding enzymes that modify anthocyanins are also upregulated: AT5MAT (AT3G29590) involved in malonation (D'Auria et al., 2007), and UF3GT/UGT79B1 (AT5G54060) involved in glycosylation (Yonekura‐Sakakibara et al., 2012). Also upregulated is the gene for GSTF12/TT19 (AT5G17220), which is likely to be involved in transport of anthocyanins into tonoplasts (Sun et al., 2012). Notably, genes encoding three TFs that upregulate many of the biosynthesis genes are themselves upregulated in *lppγ lppɛ1*: the gene for PAP1/MYB75 (AT1G56650), which upregulates *PAL* (encoding phenylalanine ammonia‐lyase), *CHS* (chalcone synthase), *F3H* (flavonoid 30‐hydroxylase), *DFR*, and *ANS/LDOX* (Tohge et al., 2005), is itself 14.6‐fold upregulated; PAP2/MYB90 (AT1G66390), which upregulates *PAL*, *CHS*, *CHI* (encoding chalcone isomerase), *DFR*, *ANS/LDOX*, and *UF3GT* (Gonzalez et al., 2008), is 159‐fold upregulated; and the gene for TT8 (AT4G09820), which acts with PAP1 to regulate DFR (Nesi et al., 2000), is 18.8‐fold upregulated. This upregulation of many biosynthesis genes in the anthocyanin pathway as well as their positive regulators may explain the 18‐fold increased level of anthocyanin pigments observed for the *lppγ lppɛ1* mutant (Figure S1). Upregulation of the genes for any one of four MYB TFs (PAP1/MYB75, PAP2/MYB90, MYB113, or MYB114) has been reported to increase anthocyanin production in young leaves and under osmotic stress, and most of the variation in anthocyanin accumulation in various lines of Arabidopsis was attributed to PAP2/MYB90 (Bac‐Molenaar et al., 2015).

### Suppressor screen in *lppγ lppɛ1* identified MED16 of the mediator complex

**Figure 3:**
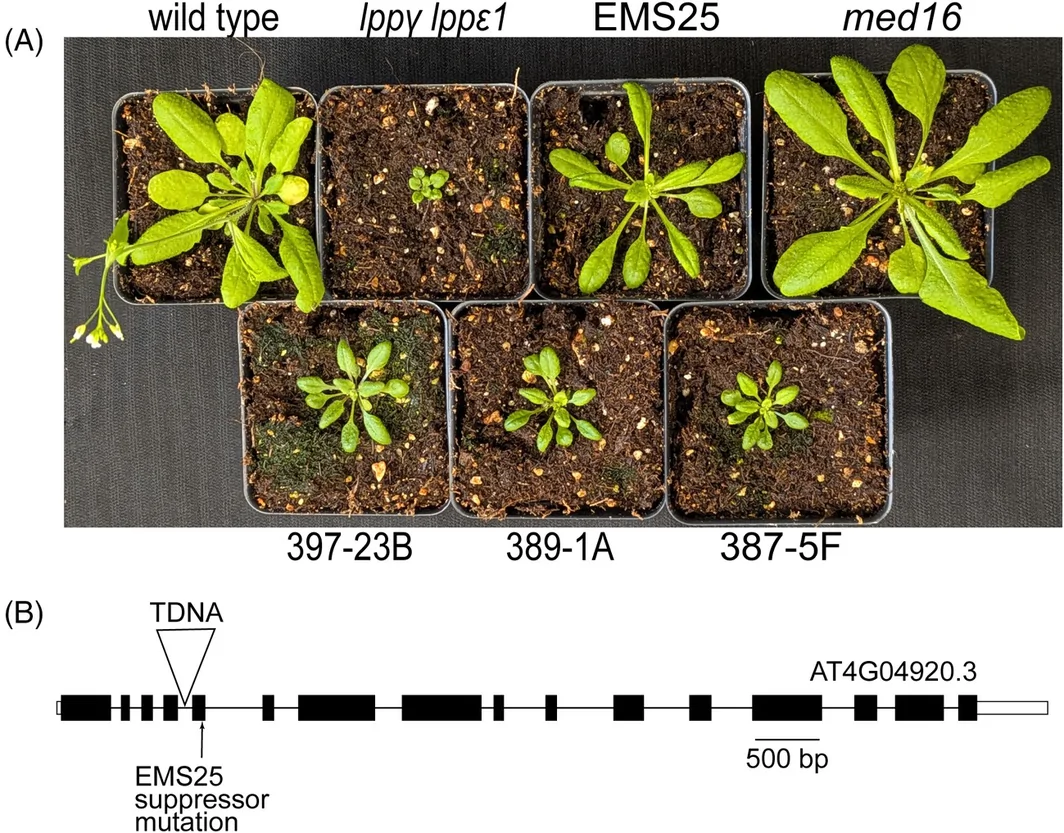
Comparison of the *lppγ lppε1* dwarf mutant to the wild type and suppressor mutant lines. (A) Comparison of representative plants of wild type, the double‐mutant *lppγ lppɛ1*, the suppressor line EMS25‐20, and the SALK *med16* mutant on the top row, and triple deletion mutant (*lppγ lppɛ med16*) lines 397‐23B, 389‐1A, and 389‐5F on the bottom row. (B) Structure of the Arabidopsis *MED16* gene showing the locations of the SALK_048091 line T‐DNA insertion and the EMS25 suppressor line point mutation. Black bars are exons, white bars are the 5″ and 3″ untranslated regions, and black lines are introns.

The result of the RNA‐Seq analysis of *lppγ lppɛ1* described above suggests a general upregulation of genes related to abiotic stress by possibly activating stress‐responsive signaling cascades leading to the stunted growth of *lppγ lppɛ1*. To test this hypothesis and identify components of the likely underlying, de‐repressed signaling pathway, we conducted a suppressor mutant screen in *lppγ lppɛ1*. EMS‐mutagenized seeds of the Arabidopsis *lppγ lppɛ1* double mutant (Cook et al., 2024) were screened by direct planting in soil (approximately 1080 seeds) or by screening on Murashige and Skoog medium (MS) plates prior to transfer to soil (approximately 13 400 seeds). Seventeen plants from soil and 18 plants from plates were identified as being larger than the *lppγ lppɛ1* parental line with two and five plants, respectively, determined to be close to wild type in size. One plant, hereafter referred to as EMS25 (the EMS‐generated suppressor mutation line), was the closest to the wild type in size, leaf shape, and color and was selfed to obtain a homozygous line that still contained both the *lppγ* and *lppɛ1* mutations. Plants of the EMS25 suppressor line were larger and lighter green in color than *lppγ lppɛ1* plants (Figure 3A).

Backcrossing of the EMS25 line to *lppγ lppɛ1* produced one small plant in the F1 population and a segregating ratio of five big plants to 18 small plants (close to 1:3) in the F2 population, suggesting that the underlying suppressor mutation represents a recessive allele. This was confirmed in an F3 population originating from a heterozygous F2 plant with 69 large and 229 small plants (χ^2^ test, *P* < 0.01). One big plant from the backcross, EMS25‐20 (progeny line from backcrossing EMS25 to the *lppγ lppɛ1* double mutant), was selfed to provide seeds for the stress tests. Both the EMS25 and the EMS25‐20 lines still contain homozygous *lppγ* and *lppɛ1* mutations, ruling out reversion of the mutation in either gene as the cause for the increase in size. The increased level of anthocyanins in the *lppγ lppɛ1* line initially reported by Cook et al. (2024) was also reversed in the EMS25‐20 line (Figure S1).

Sequencing of genomic DNA from large (homozygous for suppressor) and small (heterozygous or homozygous wild type at that locus) F3 plants identified point mutations in a region on chromosome four as the most likely cause for suppression of the small phenotype of *lppγ lppɛ1*. With the help of the SIMPLE pipeline (Wachsman et al., 2017), six candidates for the suppressor mutation on chromosome four were identified based on the large number of single‐nucleotide polymorphisms (SNPs) in these genes in the large plants (carrying the suppressor mutation) and few or absence of SNPs in the small plants (comparable to the *lppγ lppɛ1* parent), relative to the reference Col‐0 (the *Arabidopsis thaliana* wild‐type Columbia‐0 line) genome (Table S3; Figure S2). One‐point mutation was dismissed for being a downstream variant in a hypothetical protein (AT4G07666). Four candidates were missense mutations: (1) a Pro482Ser mutation in Ca‐dependent serine/threonine protein kinase 31 (CPK31; AT4G04695); (2) a Gly318Arg mutation in a basic‐leucine zipper (bZIP) family TF (AT4G06598); (3) a conservative Arg353Lys substitution in an unidentified nucleic acid binding protein (AT4G06479); and (4) a Pro532Leu mutation in a chloroplast XyG glucan synthase (CSLC12; AT4G07960). None of these genes showed significantly altered expression in the *lppγ lppɛ1* RNA‐Seq data. Based on the statistics given in Table S3, we judged the most likely candidate for the suppressor mutation to be an early stop codon (Trp231*) in the gene for subunit 16 of the Mediator complex (*MED16*; AT4G04920). As this stop codon occurred early in the coding sequence of the gene (Figure 3B), it seems unlikely that a functional MED16 protein was present in the homozygous mutant.

The Mediator complex affects RNA polymerase II‐based transcription, particularly in stress responses. In plants, MED16 specifically has been reported to be involved in ABA transcriptional responses, cold acclimation and heat stress, drought and osmotic stress, salt stress, defense pathways, anthocyanin production, and other stress responses (reviewed in Buendia‐Monreal & Gillmor, 2016; Chong et al., 2020; Yang et al., 2016). We obtained seeds of the known *med16* T‐DNA insertion mutant SALK_048091 and selfed the plants to obtain a homozygous *med16* mutant line. As previously reported (Knight et al., 2009; Liu et al., 2019; Yang et al., 2014), the *med16* plants were larger and lighter in color than wild type; they were also slightly larger than EMS25 plants but of a similar color (Figure 3A). After crossing the *lppγ lppɛ1* line with the Salk *med16* line and selfing the progeny, we obtained seven F2 plants that were homozygous mutants for *lppγ* and *lppɛ1*, and possibly homozygous for *med16*. After selfing three of these plants (398‐23B, 389‐1A, and 389‐5F), we found that all F3 offspring were homozygous triple mutants. The triple mutant offspring were all bigger than *lppγ lppɛ1* plants but not as large as the EMS25 mutant line (Figure 3A; Figure S7), presumably due to other differences in the genetic background of the EMS25 parent versus the Salk parent. However, these triple deletion plants support the hypothesis that a mutation in the *MED16* gene is responsible for suppression of the *lppγ lppɛ1* small phenotype in the EMS25‐derived plants.

In the RNA‐Seq data, we found many differentially expressed genes in the *lppγ lppɛ1* double mutant that are MED16‐influenced (Figure 2D) as reported in the cold stress data from Hemsley et al. (2014) and the ABA signaling data from Guo et al. (2021). Upregulated genes that are involved in aforementioned stress responses include *KIN1*, *KIN2/COR6.6*, *LTI30/XERO2*, and *COR413‐TM1/COR414‐TM1/DEG25*. Potentially lipid‐related genes include three genes encoding bifunctional inhibitor/lipid‐transfer protein/seed storage 2S albumin superfamily proteins; the Δ9 stearoyl‐ACP desaturase genes *AAD4* (AT3G02620) and *ADS1.2* (AT1G06090) (Kachroo et al., 2007; Smith et al., 2013); *LTP3* (AT5G59320), encoding a lipid transfer protein induced by abiotic stress, pathogens, and ABA and reported to prevent establishment of salicylic acid‐mediated plant defense (Gao et al., 2016); *LTP4* (AT5G59310), a paralog of and functionally redundant with *LTP3* (Gao et al., 2016); and *GGL14/OSP1* (AT2G04570) and *CUS2* (AT5G33370), both encoding GDSL‐motif esterases/acyltransferases/lipases involved in cuticular wax synthesis (Hong et al., 2017; Tang et al., 2020). Most of the downregulated MED16‐influenced genes were altered less than 3‐fold, with the notable exception of *LOX4* (AT1G72520), encoding a 13‐lipoxygenase induced by abiotic stresses and involved in JA synthesis (Yang et al., 2020). *MED16* itself is only upregulated by about 20% in the *lppγ lppɛ1* mutant relative to the wild type, and none of the other Mediator Complex subunit genes found in the RNA‐Seq data were significantly altered. In a known SALK *med16* mutant, the anthocyanin‐related TF gene *PAP1/MYB75* (upregulated in *lppγ lppɛ1*) has been reported to be downregulated about 3.4‐fold relative to wild type (Guo et al., 2021), which may at least partially explain the lighter green leaves in the EMS25 suppressor mutant as well as the SALK *med16* mutant (Figure 3A; Figure S1).

### The *lppγ lppɛ1* mutant is more resistant to some ABA‐associated stresses

**Figure 4:**
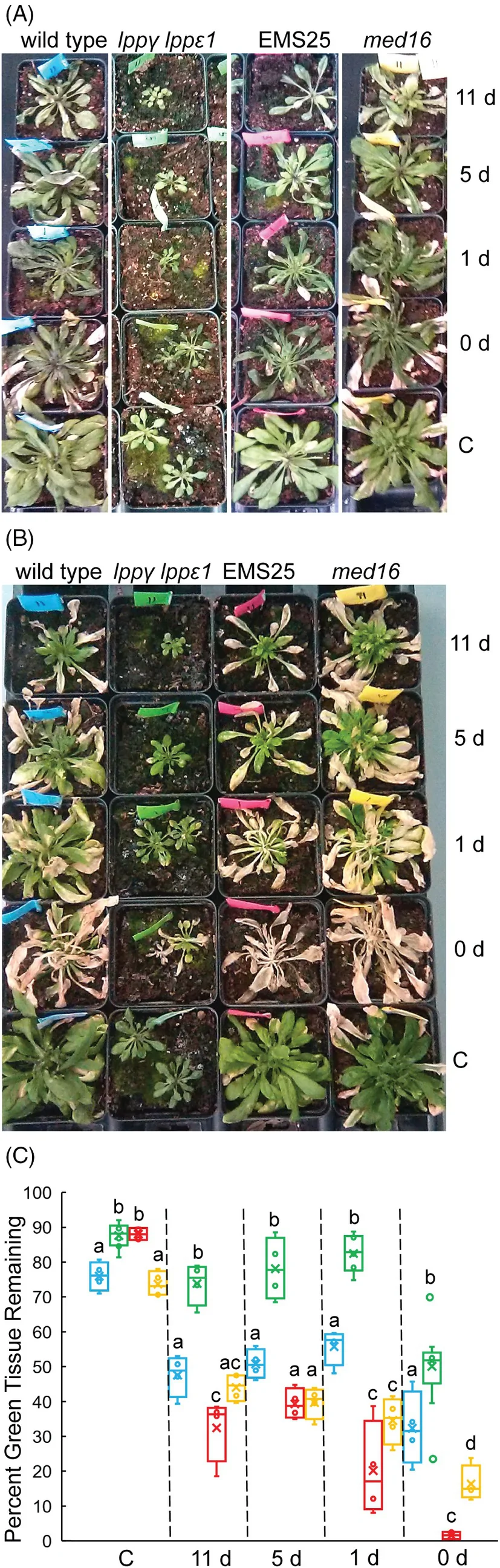
*lppγ lppɛ1* mutant shows enhanced resistance to cold stress as compared to the wild type. Plants of all four lines were acclimated at 4–6°C for 11 (top row), 5 (second row), or 1 (third row) day(s) or had no acclimation (fourth row). Plants were then frozen at −7 to 8°C for 3 nights and returned to normal growth conditions to recover. (A) Plants immediately after freezing. (B) Plants after 7 days of recovery. Plants that were never acclimated or frozen are shown for comparison (bottom row). Representative plants are shown here; all plants are shown in Figure S3. (C) Box‐and‐whisker plot of the percent of green tissue remaining after freezing followed by 7 days of recovery. Blue = wild type; green = *lppγ lppɛ1*, red = EMS25‐20, gold = *med16*. Outliers were defined as greater than Q3 + (1.5 × IQR) or less than Q1–(1.5 × IQR). *n* = 4–5 samples per plant line per days of acclimation; letters indicate similarities and differences by one‐way anova and post hoc Tukey test.

Based on the RNA‐Seq data, we tested the responses of the *lppγ lppɛ1* mutant to various stresses such as cold, drought, and salt. The *lppγ lppɛ1* mutant showed enhanced resistance to some stresses but not others. Figure 4 and Figure S2 compare the *lppγ lppɛ1* mutant to the wild type, the EMS25‐20 suppressor strain, and the SALK *med16* mutant after 0 to 11 days of cold acclimation followed by 2 nights of freezing before being returned to normal growth conditions to recover for 7 days. All plants with at least 5 days of cold acclimation survived the freezing, albeit with leaf damage. The percent of green tissue remaining was significantly higher in the *lppγ lppɛ1* mutant compared with the wild type, while that of the EMS25‐20 and *med16* lines was lower than wild type. With only 1 day of cold acclimation, all of the wild type and *lppγ lppɛ1* plants survived with at least 50% of their tissue remaining green, but the *med16* plants and EMS25‐20 plants lost about two‐thirds of their green tissue. The *med16* mutant (also called *sfr6*, SENSITIVE TO FREEZING 6) is known to be more sensitive to freezing than the wild type due to a reduction in expression of many cold‐related genes (Hemsley et al., 2014; Knight et al., 1999), and the EMS25‐20 line was expected to react similarly. Without prior cold acclimation, the wild‐type plants retained only approximately 35% of green tissue; the *med16* and EMS25‐20 plants retained approximately 15% or 2% of green tissue, respectively. However, the *lppγ lppɛ1* plants retained over 50% green tissue. These data suggest that *lppγ lppɛ1* is significantly better primed to survive freezing than the wild type. Several cold‐related genes (Figure 2A) are upregulated in the *lppγ lppɛ1* mutant; in particular, *KIN1*, *KIN2/COR6.6*, *LTI78/COR78/RD29A/LTI140*, and *COR15A*, all of which were notably downregulated in the *med16* (*sfr6*) mutant (Boyce et al., 2003; Lee et al., 2021).

**Figure 5:**
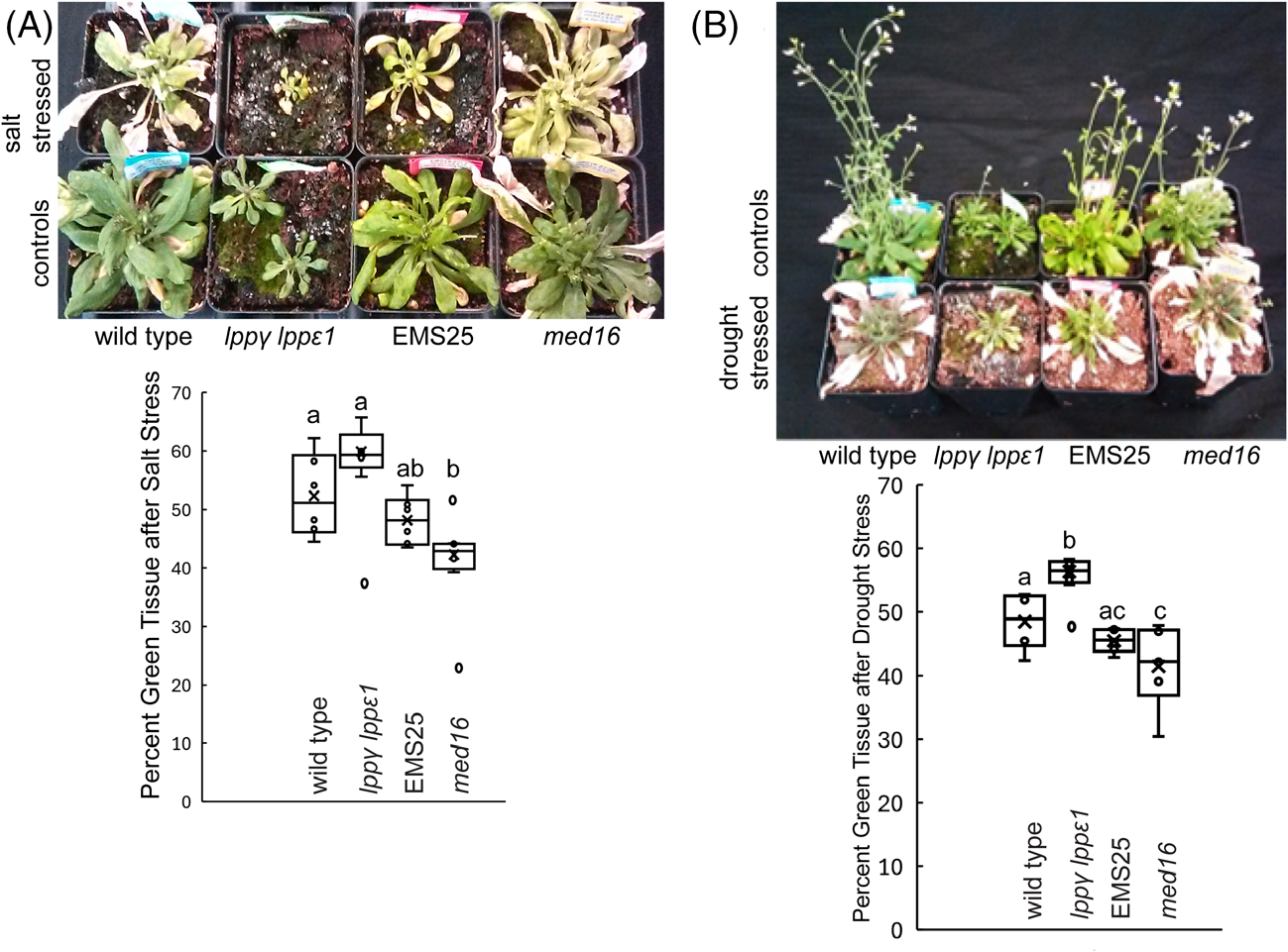
Salt and drought sensitivity of the *lppγ lppɛ1* mutant as compared to the wild type. Plants of the wild type, the *lppγ lppɛ1* mutant, the EMS25 suppressor mutant, and the *med16* mutant were watered every other day with 150 mm NaCl (A) or exposed to 22 days without water (B). Representative plants are shown here; all plants are shown in Figure S4. Shown below the photograph is a box‐and‐whisker plot of estimating the percent of remaining green tissue after stress exposure. Outliers were defined as greater than Q3 + (1.5 × IQR) or less than Q1–(1.5 × IQR); *n* = 6 samples per plant line per condition; letters indicate similarities and differences by one‐way anova and post hoc Tukey test.

**Figure 6:**
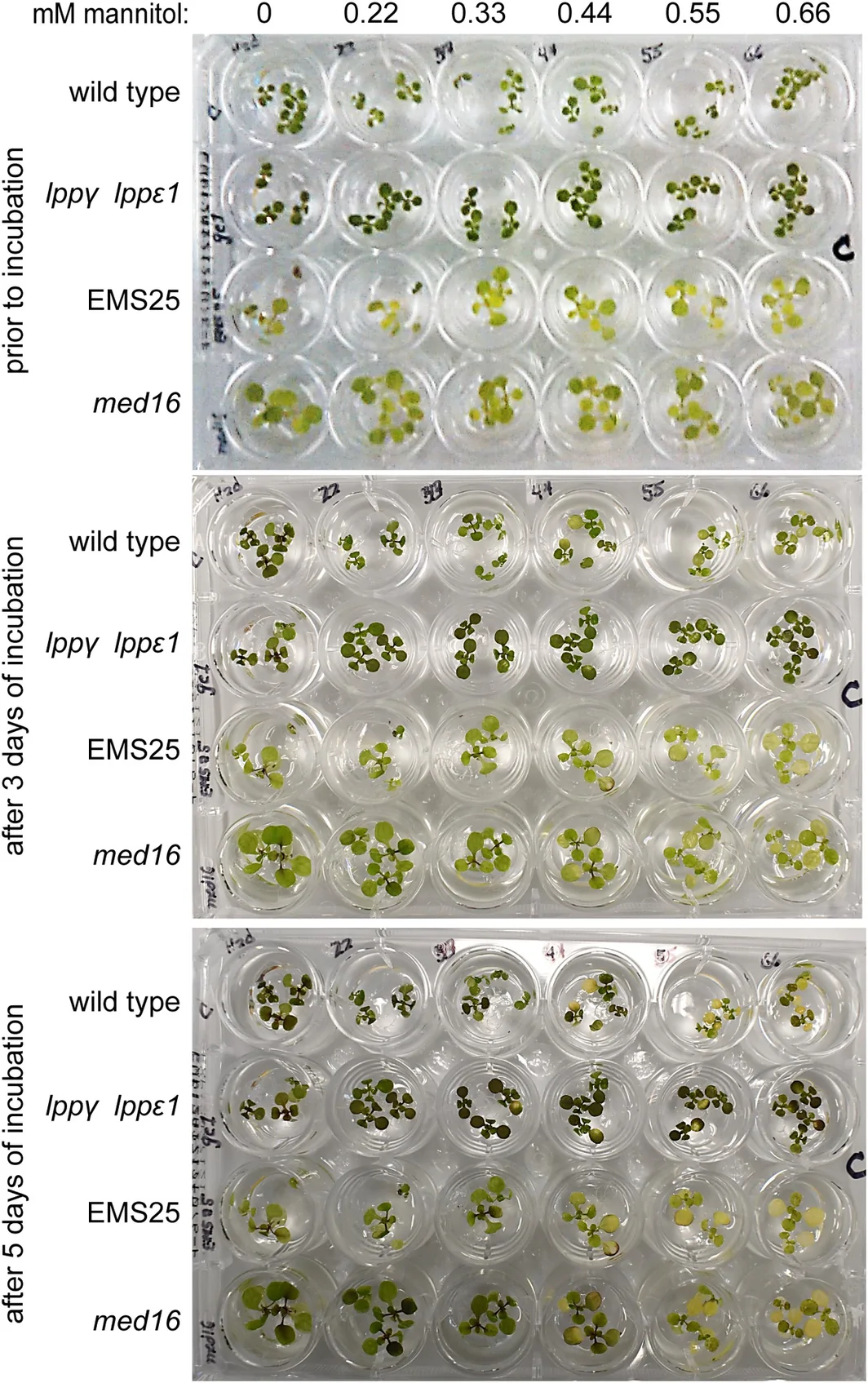
*lppγ lppɛ1* mutant is more resistant to mannitol‐generated osmotic stress than the wild type. Plants of the wild type, the *lppγ lppɛ1* mutant, the EMS25‐20 suppressor mutant, and the *med16* mutant were floated on varying concentrations of mannitol for 3 to 5 days. One of three trays is shown here; all trays are shown in Figure S5, and all data are tabulated in Table S4.

Figure 5 and Figure S3 compare the *lppγ lppɛ1* mutant to the wild type, the EMS25‐20 suppressor strain, and the SALK *med16* mutant after eight waterings with 150 mm NaCl (salt stress; Figure 5A) or 22 days without water (drought stress; Figure 5B). After being watered with saltwater, the *lppγ lppɛ1* mutant retained an average of approximately 8% more green tissue than the wild type while the green tissue level of the EMS25‐20 was similar to wild type, and the *med16* plants had slightly less green tissue than wild type; however, these differences were not significant (χ^2^ test, *P* > 0.9). After water deprivation, the wild type, EMS25‐20, and *med16* lines were comparably yellowed and dried. While the *lppγ lppɛ1* mutant retained significantly more green tissue, as might be predicted from the number of drought resistance genes that are upregulated in this mutant (Figure 2B), caution is warranted in interpreting these data: smaller plants in the same size pots compared with larger plants may use less water and may not experience the same level of water stress as larger plants. The *med16* mutant has been previously reported to be more sensitive to drought stress than the wild type (Boyce et al., 2003; Lee et al., 2021). However, in one case (Wathugala‐Gamage, 2010), sensitivity of *med16*/*sfr6* to salt and drought stress was not reproducible, and in our experiment, it also seemed mostly comparable to wild type. The EMS25 suppressor plants carrying a mutation in *MED16* looked similar to the SALK *med16* mutant plants. Thus, despite the *lppγ lppɛ1* mutant having upregulated genes related to salt and drought stress amelioration, it was not significantly more resistant to salt or drought stress than the wild type. In contrast, the *lppγ lppɛ1* mutant appeared more resistant to mannitol‐generated osmotic stress (Figure 6; Table S5). When seedlings were floated for 3 days in ≥0.44 m mannitol, the *lppγ lppɛ1* plants remained green while the cotyledons and first leaves of the wild type, EMS25‐20, and *med16* plants became chlorotic. After 5 days the *lppγ lppɛ1* cotyledons began to bleach. These results parallel those reported in (Boyce et al., 2003), who demonstrated that *med16*/*sfr6* cotyledons were very chlorotic and leaves were partly chlorotic at 0.44 m mannitol while the wild‐type cotyledons were only slightly chlorotic and leaves were still green. We found that EMS25‐20 plants, like *med16* plants, were more sensitive to this osmotic stress while *lppγ lppɛ1* plants were more resistant. It is not unreasonable that mutations affecting some part(s) of the ABA signaling pathway, such as resistance to cold, might not affect other aspects, such as osmotic stress. For example, a mutant in the ABA core signaling pathway, *abi1‐1*, was reported to be defective in SnRK2 activation by ABA but not by osmotic stress (Li, Chen, et al., 2024), and osmotic and salt stress signaling also activates more and different SnRK2s than does the ABA signaling pathway (Boudsocq et al., 2004).

**Figure 7:**
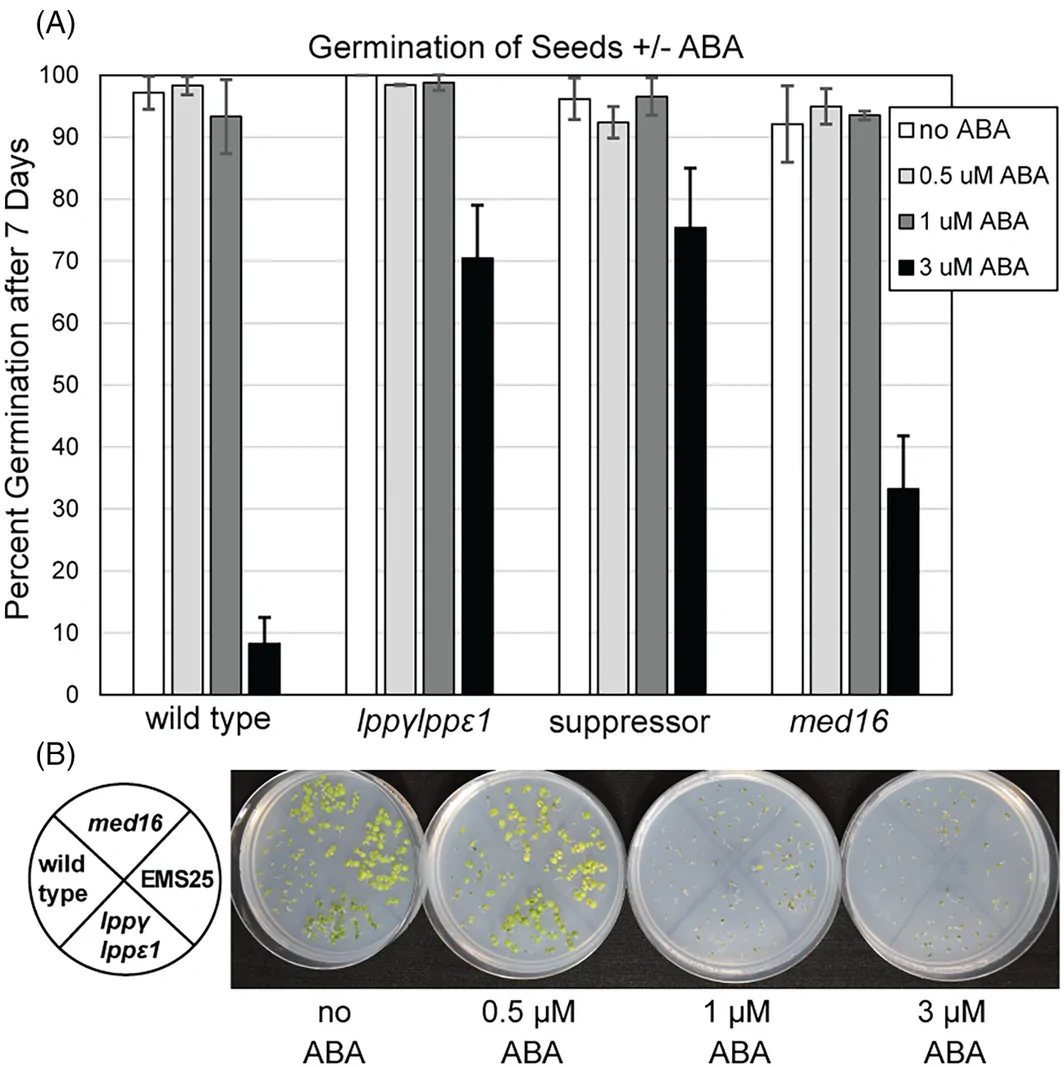
Effect of abscisic acid (ABA) on seed germination. (A) Comparison after 7 days of the percent germination of seeds from the *lppγ lppɛ1* mutant, the EMS25‐20 suppressor mutant, and the *med16* mutant to the wild type on plates containing no ABA, 0.5 μm ABA, 1 μm ABA, or 3 μm ABA. Results are averages ±SD of three experiments, each containing 40–129 seeds of each type. All data are tabulated in Table S5A. (B) Comparison of the greening of cotyledons of the four lines on varying concentrations of ABA after 10 days. The quadrants on each plate were planted as shown in the diagram. Representative plates are shown here; all plates are shown in Figure S6 and all data are tabulated in Table S5B.

Many genes related to ABA responses were differentially expressed in the *lppγ lppɛ1* mutant compared with the wild type (Tables 2 and 3). Exogenous ABA inhibits seed germination and reduces cotyledon greening (Garciarrubio et al., 1997; Jiang et al., 2025; Lopez‐Molina et al., 2001; Sajeev et al., 2024; Sun et al., 2020; Zhao et al., 2022), so the effects of ABA on seed germination and cotyledon greening were tested. The percent of seeds that germinated after 7 days was similar for the wild type, the *lppγ lppɛ1* mutant, the EMS25‐20 suppressor, and the *med16* mutant on plates without ABA or with 0.5 μm ABA (Figure 7A). However, at 1 μm ABA, the germination of *lppγ lppɛ1* seeds was noticeably less inhibited than the germination of seeds from the other lines. At 3 μm ABA, germination was severely inhibited for the wild‐type seeds, while 75–80% of *lppγ lppɛ1* and EMS25‐20 seeds germinated and approximately 35% of the *med16* mutant seeds germinated (Table S5A). The *med16* mutant has been previously reported to be less sensitive than wild type to ABA inhibition of germination (Guo et al., 2021), although it is clearly more sensitive than *lppγ lppɛ1* and EMS25‐20. When the level of cotyledon greening was compared after 10 days of growth (Figure 7B; Figure S6), nearly every seed that germinated on plates without ABA had green cotyledons; however, the EMS25‐20 and *med16* cotyledons were larger and more expanded. Cotyledon greening was slightly lower on the 0.5 μm ABA plates. On the 1 μm ABA plates, 74% of the *lppγ lppɛ1* plants, 80% of the EMS25‐20 plants, and 77% of the *med16* plants had small but green cotyledons, as compared to only 26% of the wild‐type plants. At 3 μm ABA, only 11% of wild type germinated seeds, 34% of *lppγ lppɛ1* germinated seeds, and 38–39% of EMS25‐20 and *med16* germinated seeds gave rise to green cotyledons (Table S5B). The *lppγ lppɛ1* mutant therefore seems significantly more resistant than the other lines to ABA inhibition of germination, but only slightly more resistant than wild type to ABA inhibition of further growth.

## DISCUSSION

### The *lppγ lppɛ1* mutant's stress‐response phenotype is not mediated by JA or SA

Two external attributes of the *lppγ lppɛ1* mutant suggest that it is constitutively responding to stress under ideal growth conditions: dwarfism and anthocyanin production. Mutants that overproduce the defense hormone JA show dwarfism and increased anthocyanin production (Cook, 2023; Wang et al., 2018). Production of JA is increased under biotic stresses such as pathogens, insect attack, or wounding, and increased levels of JA slow plant growth (Havko et al., 2016; Zhang & Turner, 2008). However, the JA‐dependent stress pathway does not seem to be activated in the *lppγ lppɛ1* mutant. The levels of three genes associated with JA accumulation (*VSP1*, AT5G24780; *PDF1.2*, AT5G44420; and *LOX2*, AT3G5140) were reported to be low in the mutant (Cook et al., 2024). Our RNA‐Seq data indicate that the mRNA level of *VSP1* is upregulated and the level of *PDF1.2* is downregulated in the *lppγ lppɛ1* mutant, but both are at significantly lower levels than ABA‐responsive genes (Data S1). The expression data from *LOX2* were not significant. The relatively normal leaf shape of the *lppγ lppɛ1* mutant was also unlike the short, round leaves of JA‐overproducing plants (Cook, 2023; Wang et al., 2018). Three JAZ genes (*JAZ4*, AT1G48500; *JAZ6*, AT1G72450; *JAZ12*, AT5G20900) were 1.2‐ to 2.5‐fold upregulated in the *lppγ lppɛ1* mutant, which might contribute to repression of JA signaling in this mutant; the expression of other *JAZ* genes was unchanged.

Mutants that over‐accumulate the defense hormone SA also show stunted growth (Li et al., 2022; Ortega et al., 2024). Western blot analysis indicated that the SA‐induced PR1 protein (encoded by *PR1*, AT2G14610) was not induced in the *lppγ lppɛ1* mutant, and the *lppγ lppɛ1* phenotype did not resemble that of a constitutive SA response‐activated mutant deficient in the *CPR1* gene (AT4G12560), leading to the conclusion that the SA pathway was also not activated (Cook et al., 2024). The *lppγ lppɛ1* phenotype also does not resemble a dwarf mutant deficient in the *ACD6* gene (AT4G14400) that showed constitutive SA‐mediated defense (Ng et al., 2011). Cold‐regulated genes that were downregulated in an SA‐overproducing mutant (Ortega et al., 2024) are either unchanged or upregulated in the *lppγ lppɛ1* mutant. GO categories for responses to bacteria and fungi, which are common inducers of JA or SA defense signaling, are downregulated based on the RNA‐Seq data, suggesting that the *lppγ lppɛ1* mutant could be deficient in its response to biotic stresses. Thus, the constitutive *lppγ lppɛ1* stress‐response phenotype appears to be limited to abiotic stress.

### Anthocyanin accumulation in *lppγ lppɛ1* is reversed by loss of MED16

Anthocyanin accumulation is seen under stress conditions such as cold or drought (Li & Ahammed, 2023). It has been shown that in stressed plants with sub‐optimal photosynthesis, increased levels of shikimate pathway intermediates shift the phenylpropanoid pathway toward anthocyanin production (Vogt, 2010). The *lppγ lppɛ1* mutant shows increased anthocyanin production under normal light, and it was determined that it has lower photosystem II efficiency (Cook et al., 2024); reducing the light level did lower anthocyanin but did not alter the dwarfed phenotype, suggesting that the increase in anthocyanins in *lppγ lppɛ1* is due to increased light sensitivity of the mutant, but not directly related to the growth phenotype (Cook et al., 2024). MED16 has been reported to be required for both growth and repression of phenylpropanoid biosynthesis genes in a *med5b* mutant with dwarfism and constitutive phenylpropanoid accumulation (Dolan et al., 2017). The larger size and lighter color of EMS25 suppressor line plants (*lppγ lppɛ1 med16*; Figure 3A) demonstrate that MED16 is also required for the downregulation of anthocyanin biosynthesis and enhanced growth in the suppressor mutant. These results suggest that a functional MED16 is required for suppression of excess phenylpropanoid synthesis and for normal growth under non‐stress conditions.

### ABA signaling is enhanced in *lppγ lppɛ1* but partly reversed by introducing *med16*

ABA signaling is upregulated under several forms of abiotic stress, including cold, drought, salt, or osmotic stresses. Many genes involved in ABA responses are upregulated in the *lppγ lppɛ1* mutant (Tables 2 and 3; Data S1). The mutations in *lppγ lppɛ1* seem to bypass and/or turn on the core ABA signal transduction pathway by upregulating transcription of ABA‐regulated genes in the absence of any ABA signal. The ABA‐upregulated bZIP TF‐encoding gene *ABI5*, which is itself upregulated 16‐fold in *lppγ lppɛ1*, has several targets that are also upregulated in *lppγ lppɛ1*, including *ABR* (195‐fold), *AFP3* (9.5‐fold), and *AFP1* (19‐fold) (AT1G69260; Garcia et al., 2008; Lynch et al., 2017). A pyrophosphorylase‐encoding gene *PPa2* (AT2G18230), an ABI5 target gene that was reported to be upregulated in an *ABI5* overexpressor (Chen et al., 2024), is also approximately threefold upregulated in *lppγ lppɛ1*; the RNA polymerase subunit beta‐encoding gene (AT2G41950) that is downregulated in the *ABI5* overexpressor is also approximately twofold downregulated in *lppγ lppɛ1*. This could suggest that *lppγ lppɛ1* may be overcoming normal, non‐stress repression of ABA‐related gene expression in a manner that mimics upregulation of *ABI5*, which encodes a core component of the ABA signaling pathway. On the contrary, ABA, via ABI5, is an important regulator of seed germination. Mutants deficient in ABI5 were reported to be resistant to ABA‐mediated arrest of seed germination and seedling growth, while overexpression of *ABI5* caused increased sensitivity to ABA (Zhao et al., 2020, 2022). In the case of the *lppγ lppɛ1* mutant, the *ABI5* gene is upregulated yet *lppγ lppɛ1* seeds were still more resistant to applied ABA than wild‐type seeds. However, exogenous ABA also induces the expression of genes encoding clade A protein phosphatases of type 2C (PP2Cs), which are negative regulators of ABA signaling (Zhao et al., 2020). In the absence of ABA, the ABI2 protein, whose gene expression is approximately fourfold upregulated in *lppγ lppɛ1*, dephosphorylates ABI5 to reduce its stability (Ali et al., 2024; Skubacz et al., 2016); however, the SnRk2.6 kinase whose gene expression is 2.7‐fold upregulated may be phosphorylating and activating ABI5 (Kulik et al., 2011; Nakashima et al., 2009). The *AFP1* and *AFP3* genes encoding negative regulators of ABA signaling (Vittozzi et al., 2024) are also upregulated. This tug‐of‐war among multiple players in the ABA core signaling pathway may explain why *lppγ lppɛ1* is resistant to some ABA‐mediated stresses but not all.

It is noteworthy that several genes encoding PP2Cs in the ABA signaling pathway are upregulated in the *lppγ lppɛ1* mutant (Table 2), which may help counterbalance upregulation of *ABI5*, at least under certain conditions such as seed germination. Overexpression of either of two PP2C‐encoding genes that are upregulated in the *lppγ lppɛ1 mutant, PP2Ca* (Kuhn et al., 2006) and *HAB1* (Saez et al., 2004), has been reported to cause insensitivity to exogenous ABA in seed germination, while a mutation in *abi1* (also upregulated in the *lppγ lppɛ1 mutant*) caused increased sensitivity (Saez et al., 2006). In double mutants of *abi1 hab1*, levels of several genes (encoding RAB18, RD29A, RD29b, KIN1, and P5CS1) involved in ABA and stress‐response pathways were generally less than twofold upregulated under normal conditions but 2.2‐ to 8.6‐fold upregulated by ABA treatment (Saez et al., 2006). In our RNA‐Seq data, these genes are 31‐fold, 5.5‐fold, 516‐fold, 57‐fold, and 10‐fold upregulated, respectively, under normal conditions, suggesting that, despite upregulation of several PP2C‐encoding genes, the ABA signaling pathway is enhanced in the *lppγ lppɛ1 mutant* in the absence of ABA. Involvement of some PP2C(s) in the *lppγ lppɛ1* phenotype is also supported by the reported triple mutants deficient in the PP2Cs *hab1 abi1 abi12* and *hab1 abi1 pplcA* which, like *lppγ lppɛ1*, showed a partly constitutive response to endogenous ABA and dwarfism (Rubio et al., 2009). However, the *lppγ lppɛ1* mutant appears to be more dwarfed than the two triple PP2C mutants and the triple mutants showed reduced seed germination in the presence of exogenous ABA (Rubio et al., 2009) while the *lppγ lppɛ1* mutant had better seed germination than the wild type (Figure 7; Figure S5).

The upregulation of the ABA signaling pathway genes in the absence of ABA appears to be counteracted by a *med16* mutation. MED16 was shown to be required for expression of genes with ABREs (ABA‐responsive motifs) in their promoters (Lee et al., 2021). For example, ABA‐induced upregulation of the cold‐responsive genes *KIN2*, *LTI78*, and *RAB18* (AT5G66400) was severely reduced in *med16* mutants (Lee et al., 2021). These genes are also upregulated by 3‐fold (*KIN2*), 5.5‐fold (*LTI78*), and 31‐fold (*RAB18*) in the *lppγ lppɛ1* mutant (Figure 2A; Data S1) and this mutant is cold‐resistant (Figure 4). The *med16* mutant and the EMS25‐20 suppressor mutant line carrying a *med16* mutation, however, were more sensitive to cold stress than the wild type. The roles of MED16 and MED25 were reported to be antagonistic in ABA responses (Guo et al., 2021), with MED16 promoting the ABA response while MED25 playing a negative role (Chong et al., 2020). These alternative roles suggest that the abiotic stress responses should be reduced in *med16* mutants and, given normal levels of MED25, this should counteract the stress phenotype of the *lppγ lppɛ1* mutant when a *med16* mutation is added.

### Probing the connection of ABA stress response to a potential PA buildup

Although the constitutive stress response of the *lppγ lppɛ1* mutant is clearly related to the ABA signaling pathway, it is not obvious how mutations in two chloroplast LPPs could affect that pathway. It was previously postulated that a localized accumulation of PA in the chloroplast outer envelope may be acting as a signaling molecule in *lppγ lppɛ1* (Cook et al., 2024). PA has been demonstrated to affect the phosphorylation states, activities and locations of proteins to which it binds (Kolesnikov et al., 2022), including proteins involved in the ABA pathway. PA was reported to tether the clade A PP2C ABI1 to the plasma membrane, decreasing its movement into the nucleus and thereby derepressing ABA signaling (Yao et al., 2014, 2024; Zhang et al., 2004), although it was also shown that ABI1 could also bind PA in the cytosol (Ndathe & Kato, 2024). However, the diacylglycerol kinase DGK5 (AT2G20900) and its product PA bind to the ABA synthesis enzyme ABA2 (AT1G52340) and promote its nuclear translocation (Li, Yao, et al., 2024) and suppression of ABA production (Yao et al., 2024). In the ABA signaling pathway, the clade A PP2Cs ABI1 (Zhang et al., 2004), PP2CA/AHG3 (Qu et al., 2018) and possibly also ABI2 (Ndathe & Kato, 2024) bind to and are inhibited by PA. Two SnRk2 family kinases have also been reported to bind PA: SNRK2.4/ASK1/SnRK2A (AT1G10940) (Julkowska et al., 2015) and SnRK2.10/SnRK2B (AT1G60940) (Testerink et al., 2004), although both are subgroup 1 SnRK2s that are generally considered ABA‐independent and react only to severe osmotic stress (Yuan & Zhao, 2025). It has also been demonstrated that the local presence of PA in membranes where it is produced can influence the location of its binding proteins (Yao et al., 2014). For example, PA specifically produced by phospholipase D ζ1 (AT3G16785) interacts with the MYB‐TF WER (AT5G14750; not found in our RNA‐Seq data set) and is necessary for its nuclear translocation (Yao et al., 2013).

Loss of function mutants of the ER‐located LPP2/PAP2 (AT1G15080) accumulated higher levels of PA and were hypersensitive to ABA‐mediated inhibition of germination (Katagiri et al., 2005; Nguyen & Nakamura, 2023). That the double knockout of the plastid LPPγ and LPPɛ1 (Cook et al., 2024) was more resistant to the same stress hints that the *lppγ lppɛ1* mutant is not likely to be experiencing a buildup of PA in the ER. When a bacterial diacylglycerol kinase (DAGK), which converts DAG to PA, was directed to the outer leaflet of the chloroplast outer envelope membrane (equivalent to ‘outside’ of the chloroplast) or the stroma, no phenotype was observed; however, when DAGK was directed to the inner leaflet of the outer envelope membrane and the outer leaflet of the inner envelope membrane, facing the intermembrane space, the plants were dwarfed (Muthan et al., 2013). This suggests that the dwarf phenotype of the *lppγ lppɛ1* mutant is more likely to be due to a buildup of PA in one or both of the membranes facing the chloroplast intermembrane space instead of the membranes facing the cytosol or the stroma. It also supports the idea that a very localized buildup of signaling PA in these membranes is possible.

The obvious next question to answer is whether PA accumulates in a specific leaflet of the chloroplast envelope membrane as predicted by the location of the LPPγ and LPPɛ1 phosphatases or as the result of the DAGK targeting experiment mentioned above. However, there is no physical method available at this time to probe local concentrations of PA in a leaflet using mass spectrometry, and molecular methods such as introducing PA‐sensitive fluorescent proteins may potentially disturb the local environment and PA concentration, affecting the outcome. Thus, how can one test the hypothesis that a local build‐up of PA in the envelope membranes leads to a retrograde signal affecting the expression of abiotic stress genes in the nucleus? And furthermore, what would lead to such a build‐up of PA as part of a chloroplast stress‐response mechanism?

**Figure 8:**
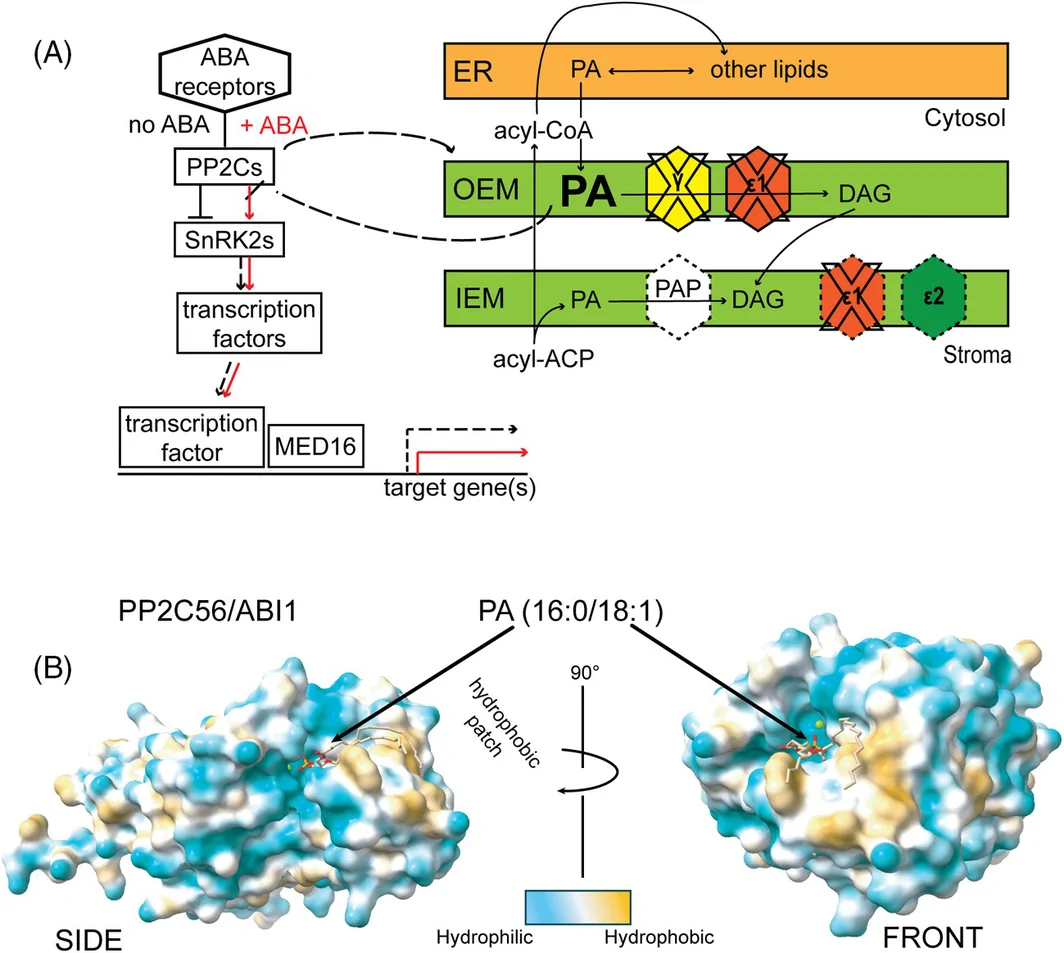
Hypothesis linking a proposed buildup of phosphatidic acid (PA) in the chloroplast outer envelope with the abscisic acid (ABA) signaling pathway (A) and structural analysis of PP2C binding to PA (B). (A) The signaling pathway is repressed by PP2Cs in the absence of ABA (black lines) but activated in the presence of ABA (red lines). In the *lppγ lppɛ1* mutant, a buildup of PA in the OEM could inhibit PP2Cs, possibly by tethering them to the chloroplast membrane, thereby relieving the repression of the SnRK2s and allowing the signaling pathway to function in the absence of ABA (dotted lines). Those upregulated ABA‐targeted genes, whose expression requires MED16, would be downregulated by a *med16* mutation. ER, endoplasmic reticulum; IEM, chloroplast inner envelope membrane; OEM, chloroplast outer envelope membrane; PAP, unidentified phosphatidic acid phosphatase in IEM; PM, plasma membrane; γ, LPPγ; ɛ1, LPPɛ1; ɛ2, LPPɛ2. Partly adapted from Cook et al. (2024). (B) Predicted structure of phosphatidic acid bound to the phosphatase PP2C56/ABI1 (At4g26080; UniProt: P49597). The interaction of phosphatidic acid [PA(16:0/18:1)] (sticks) with PP2C/ABI1 (rendered in space‐filling representation) is shown from both side and front views. The location of magnesium ions is shown as round green spheres. The acyl chains of PA are predicted to bind in a hydrophobic groove with the phosphate head group inserted into the active site, suggesting a potential mechanism for bound PA to inhibit a PP2C.

Figure 8A shows a testable scenario linking a buildup of PA in the chloroplast outer envelope with the ABA signaling pathway. In the absence of ABA (black lines), the signaling pathway is normally repressed by PP2Cs (such as ABI1, ABI2, PP2CA/AHG3, HAI1/SAG113, or HAB1) which inhibit phosphorylation of the SnRK2s (such as SnRKs 2.2, 2.3, and 2.6/OST1), which then cannot phosphorylate or activate downstream TFs (like ABI5). In the presence of ABA (red lines), the repression of the SnRK2s by the PP2Cs is relieved, so they can phosphorylate and activate downstream TFs. In the *lppγ lppɛ1* mutant, the genes encoding the two ABA core signaling PP2Cs ABI1 and ABI2, as well as the gene for PP2CA/AHG3, are upregulated in the RNA‐Seq data (Table 2A), but these PP2Cs are reported to be inhibited by binding PA (Ndathe & Kato, 2024; Qu et al., 2018), possibly by tethering the PP2C(s) to the chloroplast or other membranes (dotted lines). There is precedent for PA temporarily tethering a PP2C to the plasma membrane and thereby preventing its function (Yao et al., 2014, 2024; Zhang et al., 2004) and one instance of a PP2C (KAPP) normally being membrane‐bound (Lu et al., 2020). With the help of the Protenix Server (ByteDance AML AI4Science Team et al., 2025), we modeled ABI1 and its interaction with PA (Figure 8B). Indeed, PA can neatly bind with its phosphoryl group in the active site while the PA‐acyl chains fit into a grove near a hydrophobic patch on the surface of ABI1. This hydrophobic patch could mediate the sequestration of the protein to a membrane, and this PP2C‐membrane interaction could be facilitated by the presence of PA.

Sequestration or inhibition of PP2C(s) in the presence of PA could lead to enhanced signaling through the core ABA pathway in the absence of ABA by releasing the inhibition of SnRK2s, some of which are upregulated in *lppγ lppɛ1*. The core ABA pathway TF gene *ABI5* is also upregulated and transcription of some of the ABI5 target genes is upregulated. Since MED16 is required for the expression of some ABI5 target genes, a mutation in *MED16* might be expected to reduce the expression of these ABI5 target genes in our EMS25 suppressor line and thereby reduce the *lppγ lppɛ1* dwarf phenotype. Upregulation of genes encoding other ABA‐induced TFs such as AREBs/ABFs may also upregulate transcription of other sets of stress‐induced genes, and this may require MED16. Further experiments beyond the current work will be required to test this hypothesis more directly, and further screening of suppressor mutants will be necessary to identify additional components of the proposed PA‐based retrograde stress signaling pathway.

## MATERIALS AND METHODS

### Plant material

Mutagenesis of *Arabidopsis thaliana* mutant *lppγ lppɛ1* (Cook et al., 2024) seeds by EMS was performed by Linda Danhof as described in Kim et al. (2006). M2 seeds were stratified in 0.1% agarose at 4°C for 3 days, then sown on soil and grown for approximately 4 weeks. Plants larger than the parental *lppγ lppɛ1* line were selected. One plant (EMS25) close to the wild‐type Col‐0 in size, leaf shape, and color was selected for further study and selfed to obtain a homozygous line; this line was verified by PCR to still contain both *lppγ* and *lppɛ1* mutations by using primers from Cook et al. (2024) (Table S1 primers 1–6). EMS25 was backcrossed to the *lppγ lppɛ1* double mutant and produced one small F1 progeny plant which, upon selfing, produced a segregating population of big and small F2 plants. One big F2 plant that was homozygous for *lppγ lppɛ1*, EMS25‐20, was selfed to produce seeds for all stress experiments and its progeny were always big plants. Seeds of the SALK_048091 (*sfr6‐2*) *Arabidopsis thaliana* mutant with a T‐DNA insertion in the MED16 gene were obtained from the Arabidopsis Biological Resource Center (ABRC); one heterozygous plant was selfed to obtain a homozygous plant line for further study and verified by PCR (Table S1 primers 6–8). Triple deletion mutants (*lppγ lppɛ1med16*) were produced by crossing the *lppγ lppɛ1* mutant with the SALK *med16* mutant and selfing the F1 plants. Three putative triple mutants were selfed and all F3 offspring were also found to be triple mutants. Image J (Rasband, 1997–2018) was used to measure the relative rosette diameters of all 4‐week‐old F3 plants in the photographs (Figure S7); plant sizes in the photographs were adjusted relative to the same set of control plants in every photograph so that rosette sizes could be compared between photographs.

### Growth and stress test conditions

Plants were grown in soil (SUREMIX Professional All‐Purpose Perlite Mix; Michigan Grower Products, Inc.) at 22°C with 16 h days/8 h nights and a light intensity of 90–125 μmol m^−2^ s^−1^ for normal growth conditions. Levels of anthocyanins from approximately 10 mg of leaf tissue were measured according to (Nakata & Ohme‐Takagi, 2014). For the stress tests, plants grown for 10 days on Murashige and Skoog (MS) plates with 1% sucrose (Murashige & Skoog, 1962) were transferred to soil and grown for an additional 20 days before beginning the stress tests. For the cold stress test, plants were transferred to a cold chamber maintained at 4‐6°C with 16‐h days/8‐h nights and a light intensity of 90–125 μmol m^−2^ s^−1^ and grown for either 11, 5, or 1 day(s) of cold acclimation before freezing. For freezing treatment, the chamber temperature was set to −7 to 8°C for one 16‐h night, followed by a 12‐h day at 4–6°C, followed by a 12‐h night at −7 to 8°C, before returning to normal growth conditions (see above) for 7 days. In the salt stress test, based upon Tian et al. (2017), well‐watered plants were watered every other day for 14 days with a 150 mm NaCl solution (eight treatments) until they appeared stressed. In the drought stress test, also based on Tian et al. (2017), well‐watered plants were denied water for 22 days until they appeared stressed. To estimate the percent of green/live tissue remaining in the cold, salt, and drought stressed plants, the images in Figures S3 and S4 were cropped in UrfanView64 (a free image editing program for Windows, version 4.51) to separate individual plants and to remove non‐plant material (tags, soil, and edges of pots), then the cropped images were processed in Image J (Rasband, 1997–2018). These results were analyzed by one‐way anova with post hoc Tukey HSD test (https://astatsa.com/OneWay_Anova_with_TukeyHSD). For the mannitol‐osmotic stress test, based on Boyce et al. (2003), 11‐day‐old plants were transferred from plates to 24‐well culture dishes (three replicates) containing 2 ml of mannitol solutions (0.22 to 0.66 m) or water (control) per well and incubated under normal growth conditions (see above) for 3 to 5 days. For the ABA stress test, based on Lee et al. (2015) and Guo et al. (2021), three replicate experiments using 40–130 seeds of each line per experiment were sown on ½ MS plates with 1% sucrose and 0, 0.5 μm ABA, 1 μm ABA, or 3 μm ABA, stratified for 3 days at 4°C, and grown at 22°C; germination was scored at 7 days post‐stratification as emergence of the radicle from the seed coat, while cotyledon greening was photographed at 10 days post‐stratification.

### Genome sequencing

Genomic DNA was prepared from leaf samples from large and small plants separately from a segregating F3 population of the EMS25 line by using the Wizard Genomic DNA Purification Kit (Promega, Madison, WI, USA) and quantified by using a Qubit fluorometer (Invitrogen, Rockford, IL, USA). Samples were pooled to obtain adequate DNA at a concentration of >50 ng/μl and samples were sent to BGI for DNBseq genomic sequencing. Sequencing data were downloaded and processed by using the SIMPLE program (Wachsman et al., 2017).

### RNA library and RNA‐Seq

Total RNA was prepared from approximately 50 mg of leaf tissue from *lppγ lppɛ1* and wild‐type Col‐0 plants (three independent biological repeats) grown under normal growth conditions (see above) by using the E.Z.N.A. Plant RNA Kit (Omega BioTek, Norcross, GA, USA) and DNA contamination was removed by using the TURBO DNA‐Free kit (Invitrogen). Samples were diluted to 100 μl of 5 ng/μl and submitted for short‐read library preparation and AVITI sequencing by the Michigan State University Genomics Core (https://rtsf.natsci.msu.edu/genomics/). A list of differentially expressed genes was extracted from the RNA‐Seq data by Dr. Stephanie Hickey in the MSU Bioinfomatics Core (https:/bioinformatics.msu.edu/). Detailed RNA‐Seq methodology is available in Method S1.

### Structural analysis of ABI1 with bound phosphatidic acid (16:0/18:1)

The predicted structure of Arabidopsis PP2C56/ABI1 (At4g26080; UniProt: P49597) bound with the ligand phosphatidic acid (16:0/18:1) [PubChem CID: 5283523; 1‐palmitoyl‐2‐oleoyl‐sn‐glycero‐3‐phosphate] was determined using Protenix Server (ByteDance AML AI4Science Team et al., 2025). The top scoring structure out of four predicted was illustrated using ChimeraX (Pettersen et al., 2021). The default mapping ranges for hydrophobicity from dark cyan (hydrophilic) to white to dark goldenrod (hydrophobic) were used.

## AUTHOR CONTRIBUTIONS

CB directed this project and edited the manuscript. CH performed most of the experiments, analyzed the data, and drafted the manuscript. RC provided plant material, conceived of and supervised the suppressor screen, and edited the manuscript. YM analyzed genomic sequencing data to find the point mutations in the EMS25 suppressor mutant line and edited the manuscript. JEF provided guidance, modeled the PP2C with PA, and edited the manuscript.

## CONFLICT OF INTEREST

The authors declare no conflicts of interest.

## Supporting information


**Figure S1.** Relative anthocyanin levels.
**Figure S2.** SIMPLE plot of the LOESS ratio variable.
**Figure S3.** All plants from the cold stress test.
**Figure S4.** All plants from the salt and drought stress tests.
**Figure S5.** All trays from the osmotic stress test.
**Figure S6.** All plates from the cotyledon greening assay.
**Figure S7.** All F3 triple mutant plants with relative rosette diameters.
**Table S1.** Primers used in this study.
**Table S2.** Differentially expressed genes in *lppγ lppɛ1* with >100‐fold change relative to Col‐0 wild type.
**Table S3.** Top candidates for the suppressor of the small phenotype of *lppγ lppɛ1*.
**Table S4.** Total number counted and number of yellowed cotyledons and first leaves after mannitol stress.
**Tables S5.** (A, B) Total number of seeds, number of germinated seeds, and number of germinated seeds giving rise to greening cotyledons in the ABA test.
**Method S1.** Detailed methodology for RNA‐Seq.

**Data S1.** Excel spreadsheet of all differentially expressed genes in *lppγ lppɛ1* versus the Col‐0 wild type.

## Data Availability

The RNA‐Seq raw data discussed in this publication have been deposited in NCBI's Gene Expression Omnibus (Edgar et al., 2002) and are accessible through GEO Series accession number GSE319128 (https://www.ncbi.nlm.nih.gov/geo/query/acc.cgi?acc=GSE319128).
