# Supplementary material for: A phosphatidic acid‐based retrograde signaling pathway responsive to abiotic stress in Arabidopsis
Source: Plant J. 2026 Sep 13;127(5):e71107. doi: 10.1111/tpj.71107 (PMC13571837; doi:10.1111/tpj.71107)
Supplement: Supplementary file 1 [file TPJ-127-0-s001.docx]

**SUPPORTING INFORMATION**

**A phosphatidic acid-based retrograde signaling pathway responsive to abiotic stress in Arabidopsis.**

Carrie Hiser, Ron Cook, Yosia Mugume, John Froehlich, and Christoph Benning

**SUPPORTING METHODS**

**RNA-SEQ Data Analysis.**

Data was processed using the nf-core/rnaseq v3.18.0 pipeline built with Nextflow v24.04.4 to process and quantify transcriptomic reads using the standard defaults unless otherwise specified^1–3^. Briefly, *Salmon* v1.10.3 and *fq* v 0.12.0 were used to sub-sample FastQ files and auto-infer read strandedness ^4,5^. Adapter and quality trimming were performed using *Trim Galore!* v0.6.10 with *Cutadapt* v4.9 ^6,7^. *STAR* v2.7.11b was used to map the raw FastQ reads to the reference genome and project the alignments onto the transcriptome ^8^. The alignments were sorted and indexed using *SAMtools* v1.21 ^9^, and downstream BAM-level transcript quantification was performed with *Salmon* v1.10.3 with the --seqBias --gcBias tags ^4^.

We used *DESeq2* v1.46.0 to perform differential expression analysis ^10^. *Tximport* v1.34.0 was used to import transcript abundances and construct a gene-level *DESeqDataSet* object from *Salmon* quant.sf files ^11^. P-values were attained by the Wald test and corrected for multiple testing using the Benjamini and Hochberg method, and genes with $adjusted p-value< 0.01$ were considered differentially expressed ^12,13^.

The *fgsea* v1.32.2 was used for pre-ranked gene set enrichment analysis (GSEA), and the genes were ranked by the Wald statistic ($log2(FoldChange)/standard error$)^14^. Gene ontology enrichments were performed using the annotation file for *Arabidopsis thaliana* downloaded from [http://current.geneontology.org/products/pages/downloads.html](http://current.geneontology.org/products/pages/downloads.html%20from%20the%202025-02-06) from the 2025-02-06 release.

1. Di Tommaso, P. *et al.* Nextflow enables reproducible computational workflows. *Nat Biotechnol* 35, 316–319 (2017).
2. Ewels, P. A. *et al.* The nf-core framework for community-curated bioinformatics pipelines. *Nat Biotechnol* 38, 276–278 (2020).
3. Harshil Patel *et al.* nf-core/rnaseq: nf-core/rnaseq v3.18.0 - Lithium Lynx. Zenodo https://doi.org/10.5281/ZENODO.1400710 (2024).
4. Patro, R., Duggal, G., Love, M. I., Irizarry, R. A. & Kingsford, C. Salmon provides fast and bias-aware quantification of transcript expression. *Nat Methods* 14, 417–419 (2017).
5. Macias, M., McLeod, C. & Thrasher, A. fq (https://github.com/stjude-rust-labs/fq). Jude Children’s Research Hospital.
6. Krueger, F., James, F., Ewels, P., Afyounian, E. & Schuster-Boeckler, B. FelixKrueger/TrimGalore: v0.6.7 - DOI via Zenodo. Zenodo https://doi.org/10.5281/zenodo.5127899 (2021).
7. Martin, M. Cutadapt removes adapter sequences from high-throughput sequencing reads. *EMBnet.journal* 17, 10–12 (2011).
8. Dobin, A. *et al.* STAR: ultrafast universal RNA-seq aligner. *Bioinformatics* 29, 15–21 (2013).
9. Li, H. *et al.* The Sequence Alignment/Map format and SAMtools. *Bioinformatics* 25, 2078–9 (2009).
10. Love, M. I., Huber, W. & Anders, S. Moderated estimation of fold change and dispersion for RNA-seq data with DESeq2. *Genome Biol* 15, 550 (2014).
11. Soneson, C., Love, M. I. & Robinson, M. D. Differential analyses for RNA-seq: transcript-level estimates improve gene-level inferences. Preprint at https://doi.org/10.12688/f1000research.7563.2 (2016).
12. Wald, A. Tests of statistical hypotheses concerning several parameters when the number of observations is large. *Trans. Amer. Math. Soc.* 54, 426–482 (1943).
13. Benjamini, Y. & Hochberg, Y. Controlling the False Discovery Rate: A Practical and Powerful Approach to Multiple Testing. *Journal of the Royal Statistical Society: Series B (Methodological)* 57, 289–300 (1995).
14. Korotkevich, G. *et al.* *Fast Gene Set Enrichment Analysis*. http://biorxiv.org/lookup/doi/10.1101/060012 (2016) doi:10.1101/060012.

**SUPPORTING TABLES**

**Supporting Table S1**: Primers used in this study.

| **Primer** | | **Primer Sequence** | **Description** | **Reference** |
| --- | --- | --- | --- | --- |
| 1 | CCATTGAAGAAGCTTGAGCAC | | lppγ-1 genotyping left primer | a |
| 2 | ACCAACTCGCACCAACAATAC | | lppγ-1 genotyping right primer | a |
| 3 | TCTGTTGATACCAGAGGTGGC | | lppε1 genotyping left primer | a |
| 4 | GGATCGATTTCGAATTCTGCT | | lppε1 genotyping right primer | a |
| 5 | CCTTTTCAGAAATGGATAAATAGCCTTGCTTCC | | LB1 genotyping border primer | a |
| 6 | ATTTTGCCGATTTCGGAAC | | LBb1.3 genotyping border primer | a |
| 7 | TTGTACCAGAACTTGGCTTGG | | left primer for Salk_048091 (med16) | this work |
| 8 | CTCATCTCTTGCAGCTCCAAG | | right primer for Salk_048091 (med16) | this work |

^a^Cook R, Froehlich JE, Yang Y, Korkmaz I, Kramer DM, and Benning C. Chloroplast phosphatases LPPγ and LPPɛ1 facilitate conversion of extraplastidic phospholipids to galactolipids. Plant Physiol 2024:195(2):1506-1520. DOI: 10.1093/plphys/kiae100

**Supporting Table S2**: Differentially expressed genes in the *lppγ lppɛ1* mutant with >100-fold change relative to the wild type.

| **GeneID** | **Symbol** | **Fold**  **Change** | **Padj** | **protein encoded by gene** |
| --- | --- | --- | --- | --- |
| AT1G52690 | *LEA7* | 13,942 | 2.77E-26 | Late Embryogenesis Abundant 7; intrinsically disordered protein that stabilizes lactate dehydrogenase during drying and freezing |
| AT2G47770 | *TSPO* | 990 | 9.89E-15 | related to the bacterial outer membrane tryptophan-rich sensory protein (TspO) and the mammalian mitochondrial 18 kDa Translocator Protein; induced by osmotic or salt stress or ABA treatment |
| AT1G64110 | *DAA1* | 865 | 8.53E-113 | See Table 1 |
| AT4G08570 | *HIPP24/ ATHMP33* | 587 | 2.58E-12 | Heavy metal transport/detoxification superfamily protein |
| AT5G52300 | *LTI65/ RD29B* | 516 | 1.45E-128 | See Table 1 |
| AT3G29590 | *AT5MAT* | 466 | 6.55E-12 | a malonyl-CoA:anthocyanidin 5-O-glucoside-6"-O-malonyltransferase involved in the malonylation of anthocyanins |
| AT1G22990 | *HIPP22* | 345 | 2.81E-14 | Heavy metal transport/detoxification superfamily protein |
| AT5G42800 | *DFR* | 254 | 7.85E-50 | dihydroflavonol reductase; converts dihydroquercetin to leucocyanidin in synthesis of anthocyanins |
| AT1G21890 | *UMAMIT19* | 233 | 7.58E-07 | Usually Multiple Acids Move In And Out Transporters 19; nodulin MtN21-like transporter family protein |
| AT3G28300 | *AT14A* | 197 | 0.003852036 | a protein with sequence similarity to integrins; Note that there are two AT14A genes. |
| AT3G02480 | *ABR* | 195 | 7.98E-08 | ABA-Response Protein; LEA protein up-regulated by ABA, NaCl and mannitol,and light deprivation; expression regulated by ABI5 |
| AT2G37870 | AT2G37870 | 167 | 4.08E-62 | Bifunctional inhibitor/lipid-transfer protein/seed storage 2S albumin superfamily protein |
| AT2G21820 | AT2G21820 | 162 | 8.37E-15 | seed maturation protein |
| AT1G66390 | *MYB90/*  *PAP2* | 159 | 5.70E-07 | MYB Domain Protein 90;Production Of Anthocyanin Pigment 2 |
| AT3G53980 | AT3G53980 | 147 | 8.14E-62 | Bifunctional inhibitor/lipid-transfer protein/seed storage 2S albumin superfamily protein |
| AT1G80090 | *CBSX4* | 149 | 5.89E-06 | Cystathionine beta-synthase family protein |
| AT1G23070 | AT1G23070 | 132 | 2.16E-06 | organic solute transporter ostalpha protein |
| AT3G17520 | *LEA30* | 128 | 1.82E-06 | LEA protein family protein |
| AT4G37410 | *CYP81F4* | 127 | 1.19E-05 | cytochrome P450, family 81, subfamily F, polypeptide 4 |
| AT5G40790 | *AITR3, DIL2* | 115 | 1.81E-17 | transcription repressor that acts as feedback regulator in ABA signalling |
| AT3G24750 | *LZY5* | 109 | 6.58E-06 | LAZY5; member of the LAZY gene family |
| AT2G18550 | *HB21* | 109 | 7.72E-06 | homeobox protein 2, a homeodomain leucine zipper class I protein |
| AT3G49760 | *bZIP5* | 104 | 6.28E-06 | basic leucine-zipper 5 |
| AT3G27040 | AT3G27040 | 102 | 2.12E-05 | MATH domain/coiled-coil protein |
| AT4G11460 | *CRK30* | -130 | 3.13E-08 | a cysteine-rich receptor-like protein kinase |
| AT5G44420 | *PDF1.2/ LCR77* | -183 | 4.27E-08 | an ethylene- and jasmonate-responsive plant defensin |
| AT1G14540 | *PER4* | -199 | 6.58E-12 | Peroxidase 4; Class III peroxidase cell wall-targeted protein |
| AT4G11470 | *CRK31* | -265 | 1.06E-06 | a cysteine-rich receptor-like protein kinase. |

**Supporting Table S3:** Top candidates on chromosome four for the suppressor of the small phenotype of the *lppγ lppɛ1* mutant, as determined by the SIMPLE^b^ program. The reference genome is Col-0. EMS mut.ref, number of reads in which the large putative suppressor mutant DNA matches the reference, ideally zero; EMS mut.alt, number of reads in which the putative suppressor mutant DNA does not match the reference, ideally a large number; EMS wt.ref, number of reads in which the small *lppγ lppɛ1* parent-like plant DNA matches the reference, ideally a large number; EMS wt.alt, number of reads in which the small *lppγ lppɛ1* parent-like plant DNA does not match the reference, ideally zero or a small number.

| **Position** | **Gene symbol** | **GeneID** | **CDS**  **change** | **Protein**  **change** | **EMS**  **mut.ref** | **EMS**  **mut.alt** | **EMS**  **wt.ref** | **EMS**  **wt.alt** | **Protein function** |
| --- | --- | --- | --- | --- | --- | --- | --- | --- | --- |
| 2381645 | *CPK31* | AT4G04695 | 1444C>T  missense variant | P482S | 1 | 59 | 37 | 12 | Ca-dependent  protein kinase 31 |
| 2498984 | *MED16* | AT4G04920 | 693G>A  Stop  gained | W231* | 0 | 46 | 39 | 11 | Mediator complex  subunit 16 |
| 3068714 | AT4G06479 | AT4G06479 | 1058G>A  missense variant | R353K | 3 | 52 | 32 | 14 | nucleic acid  binding protein |
| 3665321 | AT4G06598 | AT4G06598 | 952G>A  missense variant | G318R | 1 | 44 | 44 | 19 | bZIP  transcription  factor |
| 4456326 | AT4G07666 | AT4G07666 | *376C>T  downstream variant |  | 1 | 57 | 47 | 19 | Hypothetical  protein |
| 4803133 | *CSLC12* | AT4G07960 | 1595C>T  missense variant | P532L | 4 | 50 | 36 | 11 | XyG glucan  synthase |

^b^Wachsman G, Modliszewski JL, Valdes M, Benfey PN (2017) A SIMPLE Pipeline for Mapping Point Mutations. Plant Physiol 174: 1307-1313

**Supporting Table S4:** Total number of cotyledons and first leaves counted and number of cotyledons and first leaves that were yellowed after plants were floated for 3 days or 5 days in the indicated levels of mannitol or a water-only control (supports Figure 6).

|  | | **3 DAYS** | | | | | | | | | **5 DAYS** | | | | | | | |  |
| --- | --- | --- | --- | --- | --- | --- | --- | --- | --- | --- | --- | --- | --- | --- | --- | --- | --- | --- | --- |
|  | | **PLATE A** | | | **PLATE B** | | | **PLATE C** | | | **PLATE A** | | **PLATE B** | | | **PLATE C** | | |  |
| **M mannitol** | **line** | | total | yellowed | | total | yellowed | | total | yellowed | total | yellowed | | total | yellowed | | total | yellowed | |
| 0 | wild type | | 12 | 0 (0%) | | 12 | 0 (0%) | | 12 | 0 (0%) | 12 | 0 (0%) | | 12 | 2 (17%) | | 12 | 0 (0%) | |
|  | *lppγ lppɛ1* | | 9 | 0 (0%) | | 12 | 0 (0%) | | 12 | 0 (0%) | 9 | 0 (0%) | | 12 | 0 (0%) | | 12 | 0 (0%) | |
|  | EMS25-20 | | 8 | 0 (0%) | | 8 | 0 (0%) | | 8 | 0 (0%) | 8 | 0 (0%) | | 8 | 1 (12%) | | 8 | 0 (0%) | |
|  | *med16* | | 8 | 0 (0%) | | 12 | 0 (0%) | | 8 | 0 (0%) | 8 | 0 (0%) | | 12 | 1 (8%) | | 8 | 0 (0%) | |
| 0.22 M | wild type | | 12 | 0 (0%) | | 12 | 0 (0%) | | 12 | 0 (0%) | 12 | 0 (0%) | | 12 | 0 (0%) | | 12 | 0 (0%) | |
|  | *lppγ lppɛ1* | | 9 | 0 (0%) | | 12 | 0 (0%) | | 12 | 0 (0%) | 9 | 0 (0%) | | 12 | 0 (0%) | | 12 | 0 (0%) | |
|  | EMS25-20 | | 8 | 0 (0%) | | 8 | 0 (0%) | | 6 | 0 (0%) | 8 | 0 (0%) | | 8 | 0 (0%) | | 6 | 0 (0%) | |
|  | *med16* | | 8 | 0 (0%) | | 8 | 0 (0%) | | 12 | 0 (0%) | 8 | 1 (12%) | | 8 | 0 (0%) | | 12 | 0 (0%) | |
| 0.33 M | wild type | | 12 | 2 (17%) | | 8 | 0 (0%) | | 11 | 0 (0%) | 12 | 2 (17%) | | 8 | 0 (0%) | | 11 | 0 (0%) | |
|  | *lppγ lppɛ1* | | 9 | 0 (0%) | | 12 | 0 (0%) | | 12 | 0 (0%) | 9 | 0 (0%) | | 12 | 0 (0%) | | 12 | 1 (8%) | |
|  | EMS25-20 | | 8 | 1 (12%) | | 8 | 0 (0%) | | 8 | 0 (0%) | 8 | 3 (38%) | | 8 | 0 (0%) | | 8 | 0 (0%) | |
|  | *med16* | | 8 | 2 (25%) | | 12 | 0 (0%) | | 8 | 0 (0%) | 8 | 2 (25%) | | 12 | 0 (0%) | | 8 | 0 (0%) | |
| 0.44 M | wild type | | 12 | 3 (25%) | | 12 | 4 (33%) | | 12 | 4 (33%) | 12 | 5 (42%) | | 12 | 4 (33%) | | 12 | 4 (33%) | |
|  | *lppγ lppɛ1* | | 9 | 0 (0%) | | 12 | 0 (0%) | | 12 | 1 (8%) | 9 | 0 (0%) | | 12 | 0 (0%) | | 12 | 1 (8%) | |
|  | EMS25-20 | | 8 | 3 (38%) | | 8 | 2 (25%) | | 8 | 1 (12%) | 8 | 7 (88%) | | 8 | 3 (38%) | | 8 | 3 (38%) | |
|  | *med16* | | 8 | 6 (75%) | | 12 | 1 (8%) | | 8 | 2 (50%) | 8 | 6 (75%) | | 12 | 3 (25%) | | 8 | 3 (38%) | |
| 0.55 M | wild type | | 12 | 4 (33%) | | 8 | 4 (50%) | | 10 | 3 (33%) | 12 | 8 (67%) | | 8 | 4 (50%) | | 10 | 5 (50%) | |
|  | *lppγ lppɛ1* | | 9 | 2 (12%) | | 12 | 1 (8%) | | 12 | 2 (17%) | 9 | 2 (22%) | | 12 | 1 (8%) | | 12 | 2 (17%) | |
|  | EMS25-20 | | 8 | 6 (75%) | | 6 | 2 (33%) | | 8 | 3 (38%) | 8 | 6 (75%) | | 6 | 2 (33%) | | 8 | 3 (38%) | |
|  | *med16* | | 8 | 6 (75%) | | 12 | 2 (17%) | | 12 | 3 (25%) | 8 | 6 (75%) | | 12 | 2 (17%) | | 12 | 6 (50%) | |
| 0.66 M | wild type | | 12 | 6 (50%) | | 12 | 6 (50%) | | 11 | 5 (46%) | 12 | 8 (67%) | | 12 | 6 (50%) | | 11 | 5 (46%) | |
|  | *lppγ lppɛ1* | | 9 | 2 (22%) | | 12 | 0 (0%) | | 16 | 0 (0%) | 9 | 2 (22%) | | 12 | 2 (17%) | | 16 | 0 (0%) | |
|  | EMS25-20 | | 8 | 6 (75%) | | 8 | 4 (50%) | | 6 | 4 (67%) | 8 | 6 (75%) | | 8 | 4 (50%) | | 6 | 4 (67%) | |
|  | *med16* | | 8 | 6 (75%) | | 10 | 5 (50%) | | 12 | 8 (67%) | 8 | 6 (75%) | | 10 | 5 (50%) | | 12 | 6 (50%) | |

**Supporting Table S5A:** Total number of seeds and number of germinated seeds in each of the three replicates of the ABA seed germination test (supports Figure 7A).

| **uM**  **ABA** | **Plant line** | **Total seeds tested in**  **Expt. 1** | **Germinated seeds in Expt. 1** | **Total seeds tested in Expt. 2** | **Germinated seeds in Expt. 2** | **Total seeds tested in Expt. 3** | **Germinated seeds in Expt. 3** | **Total seeds tested** | **Total germinated seeds** |
| --- | --- | --- | --- | --- | --- | --- | --- | --- | --- |
| 0 | wild type | 60 | 58 (97%) | 76 | 72 (95%) | 70 | 70 (100%) | 206 | 200 (97±3%) |
|  | *lppγ lppɛ1* | 59 | 59 (100%) | 63 | 63 (100%) | 77 | 77 (100%) | 199 | 199 (100±0%) |
|  | EMS25-20 | 51 | 51 (100%) | 50 | 47 (94%) | 108 | 102 (94%) | 209 | 200 (96±3%) |
|  | *med16* | 53 | 50 (94%) | 63 | 61 (97%) | 94 | 80 (85%) | 210 | 191 (92±6%) |
| 0.5 | wild type | 43 | 43 (100%) | 80 | 78 (98%) | 77 | 75 (97%) | 200 | 196 (98±1%) |
|  | *lppγ lppɛ1* | 59 | 58 (98%) | 67 | 66 (98%) | 122 | 120 (98%) | 248 | 244 (98±0%) |
|  | EMS25-20 | 42 | 40 (95%) | 47 | 43 (91%) | 104 | 94 (90%) | 193 | 177 (92±3%) |
|  | *med16* | 46 | 45 (98%) | 77 | 75 (97%) | 73 | 67 (92%) | 196 | 186 (95±3%) |
| 1 | wild type | 45 | 40 (89%) | 80 | 71 (89%) | 84 | 84 (100%) | 209 | 196 (93±6%) |
|  | *lppγ lppɛ1* | 49 | 49 (100%) | 79 | 77 (97%) | 87 | 86 (99%) | 215 | 212 (99±1%) |
|  | EMS25-20 | 37 | 36 (97%) | 40 | 38 (95%) | 121 | 121 (100%) | 198 | 194 (97±3%) |
|  | *med16* | 48 | 40 (83%) | 67 | 63 (94%) | 82 | 76 (93%) | 197 | 184 (93±1%) |
| 3 | wild type | 61 | 3 (5%) | 77 | 10 (13%) | 87 | 6 (7%) | 225 | 19 (8±4%) |
|  | *lppγ lppɛ1* | 46 | 30 (65%) | 71 | 57 (80%) | 94 | 62 (66%) | 211 | 149 (70±8%) |
|  | EMS25-20 | 38 | 28 (74%) | 56 | 48 (86%) | 129 | 86 (67%) | 223 | 162 (75±10%) |
|  | *med16* | 53 | 14 (26%) | 63 | 29 (46%) | 99 | 30 (30%) | 215 | 71 (33±9%) |

**Supporting Table S5B:** Total number of germinated seeds and number of germinated seeds giving rise to greening cotyledons in each of the three replicates of the ABA seed germination test (supports Figure 7B).

| **uM**  **ABA** | **Plant line** | **Germinated seeds in Expt. 1** | **Greening**  **cotyledons in Expt. 1** | **Germinated seeds in Expt. 2** | **Greening**  **cotyledons in Expt. 2** | **Germinated seeds in Expt. 3** | **Greening**  **cotyledons in Expt. 3** | **Total germinated seeds** | **Total**  **Greening**  **cotyledons** |
| --- | --- | --- | --- | --- | --- | --- | --- | --- | --- |
| 0 | wild type | 58 | 58 (100%) | 72 | 72 (100%) | 70 | 69 (99%) | 200 | 200 (100%) |
|  | *lppγ lppɛ1* | 59 | 56 (95%) | 63 | 63 (100%) | 77 | 76 (99%) | 199 | 195 (98%) |
|  | EMS25-20 | 51 | 50 (98%) | 47 | 47 (100%) | 102 | 95 (93%) | 200 | 192 (96%) |
|  | *med16* | 50 | 50 (100%) | 61 | 60 (98%) | 80 | 80 (100%) | 191 | 190 (99%) |
| 0.5 | wild type | 43 | 41 (95%) | 78 | 78 (100%) | 75 | 73 (97%) | 196 | 192 (98%) |
|  | *lppγ lppɛ1* | 58 | 56 (97%) | 66 | 61 (92%) | 120 | 101 (84%) | 244 | 218 (89%) |
|  | EMS25-20 | 40 | 40 (100%) | 43 | 43 (100%) | 94 | 69 (73%) | 177 | 152 (86% |
|  | *med16* | 45 | 45 (100%) | 75 | 72 (96%) | 67 | 60 (90%) | 186 | 177 (95%) |
| 1 | wild type | 40 | 7 (18%) | 71 | 22 (31%) | 84 | 22 (26%) | 196 | 51 (26%) |
|  | *lppγ lppɛ1* | 49 | 29 (59%) | 77 | 73 (95%) | 86 | 55 (64%) | 212 | 157 (74%) |
|  | EMS25-20 | 36 | 31 (86%) | 38 | 38 (100%) | 121 | 88 (73%) | 194 | 157 (81%) |
|  | *med16* | 40 | 29 (72%) | 63 | 54 (86%) | 76 | 59 (78%) | 184 | 142 (77%) |
| 3 | wild type | 3 | 0 (0%) | 10 | 0 (0%) | 6 | 2 (33%) | 19 | 2 (11%) |
|  | *lppγ lppɛ1* | 30 | 0 (0%) | 57 | 16 (28%) | 62 | 18 (29%) | 149 | 34 (23%) |
|  | EMS25-20 | 28 | 1 (4%) | 48 | 22 (46%) | 86 | 39 (45%) | 162 | 62 (38%) |
|  | *med16* | 14 | 1 (7%) | 29 | 8 (26%) | 30 | 19 (63%) | 71 | 28 (39%) |

**SUPPORTING FIGURES**

**Supporting Figure S1:** Relative anthocyanin levels in all four plant lines. Anthocyanin levels were calculated according to Nakata and Ohme-Takagi^c^. Three plants of each type were measured. One-way ANOVA analysis showed that the level of anthocyanins in *lppγ lppɛ1* was significantly different from wild type (p < 0.001). Anthocyanin levels in the EMS25-20 line were only significantly different from the wild type at the p<0.05 level, while the levels of the Salk *med16* line were not different.

^c^Nakata M, Ohme-Takagi M (2014) Quantification of Anthocyanin Content. Bio-protocol 4 (7) e1098.

**Supporting Figure S2:** SIMPLE plot^d^ of the LOESS (locally estimated scatter plot smoothing) ratio variable, which indicates the probability for the causal mutation for the suppressor phenotype versus chromosome location. The top candidates for the suppressor mutation are on chromosome four.

^d^Wachsman G, Modliszewski JL, Valdes M, Benfey PN (2017) A SIMPLE Pipeline for Mapping Point Mutations. Plant Physiol 174: 1307-1313

**Supporting Figure S3.** All plants from the cold stress test after 7 days of recovery. Representative plants are shown in Figure 4.

**Supporting Figure S4.** All plants from the salt stress test (A) and the drought stress test (B). Representative plants are shown in Figure 5.

**Supporting Figure S5.** All trays from the osmotic stress test. One representative tray is shown in Figure 6.

**Supporting Figure S6**: All plates from the cotyledon greening assay +/- ABA after 10 days. Representative plates are shown in Figure 7B.

**Supporting Figure S7**: All F3 plants tested that originated from from the *lppγ lppɛ1* x *med16* crosses. (A) All plants grown and measured; representative plants are shown in Figure 3A. Note that each tray of triple mutant plants has the same representative control plants on the upper left side. (B) Box-and-whisker plot of the relative rosette diameters (in pixels, as determined in Image J and adjusted for changes in the control plants’ sizes between flats). Blue = wild type; green = lppγ lppɛ1, red = EMS25-20, gold = med16. The three triple mutant strains are shown in purple (389-5F), brown (389-1A), and 397-23B (rose). Outliers were defined as greater than Q3+(1.5 x IQR) or less than Q1–(1.5 x IQR). Letters indicate similarities and differences by one-way ANOVA and post-hoc Tukey test.
